# Deciphering intratumoral heterogeneity using integrated clonal tracking and single-cell transcriptome analyses

**DOI:** 10.1038/s41467-021-26771-1

**Published:** 2021-11-11

**Authors:** Humberto Contreras-Trujillo, Jiya Eerdeng, Samir Akre, Du Jiang, Jorge Contreras, Basia Gala, Mary C. Vergel-Rodriguez, Yeachan Lee, Aparna Jorapur, Areen Andreasian, Lisa Harton, Charles S. Bramlett, Anna Nogalska, Gang Xiao, Jae-Woong Lee, Lai N. Chan, Markus Müschen, Akil A. Merchant, Rong Lu

**Affiliations:** 1 Department of Stem Cell Biology and Regenerative Medicine, Eli and Edythe Broad Center for Regenerative Medicine and Stem Cell Research, Keck School of Medicine, University of Southern California, Los Angeles, CA 90033 USA; 2 Division of Hematology, USC Norris Comprehensive Cancer Center, Keck School of Medicine, University of Southern California, Los Angeles, CA 90033 USA; 3 Center of Molecular and Cellular Oncology, Yale Cancer Center, Yale University, New Haven, CT 06511 USA; 4 Department of Immunobiology, Yale University, New Haven, CT 06511 USA; 5 Division of Hematology and Cellular Therapy, Cedars-Sinai Medical Center, Los Angeles, CA 90048 USA

**Keywords:** Cancer models, Acute lymphocytic leukaemia, Tumour heterogeneity

## Abstract

Cellular heterogeneity is a major cause of treatment resistance in cancer. Despite recent advances in single-cell genomic and transcriptomic sequencing, it remains difficult to relate measured molecular profiles to the cellular activities underlying cancer. Here, we present an integrated experimental system that connects single cell gene expression to heterogeneous cancer cell growth, metastasis, and treatment response. Our system integrates single cell transcriptome profiling with DNA barcode based clonal tracking in patient-derived xenograft models. We show that leukemia cells exhibiting unique gene expression respond to different chemotherapies in distinct but consistent manners across multiple mice. In addition, we uncover a form of leukemia expansion that is spatially confined to the bone marrow of single anatomical sites and driven by cells with distinct gene expression. Our integrated experimental system can interrogate the molecular and cellular basis of the intratumoral heterogeneity underlying disease progression and treatment resistance.

## Abstract (web summary)

DNA barcoding is a promising technology for the simultaneous analysis of genetic and phenotypic heterogeneity. Here, the authors combine DNA barcoding and single-cell RNA-seq to study heterogeneity, progression and response to therapy in B-cell acute lymphoblastic leukaemia patient-derived xenografts.

## Introduction

Cancer is a dynamic disease driven by continuous genetic and epigenetic changes^1–4^. The accumulation of these molecular alterations generates tremendous intratumoral heterogeneity^1–3,5,6^. Consequently, individual cancer cells differentially proliferate, selectively metastasize, and sporadically escape therapeutic treatment^1–3,5,6^. Cellular heterogeneity has arisen as a major hurdle in cancer treatment^7–9^. Identifying the genes underlying the heterogeneous behaviors of individual cancer cells is critically important to improving the efficacy of cancer treatment.

Recent advances in single-cell genomic and transcriptomic sequencing have greatly improved the detection of intratumoral heterogeneity at the molecular level^10–12^. However, it remains difficult to relate the molecular profiles generated by these technologies to the cellular behaviors underlying disease progression and relapse. Some studies have managed to link a few genes using massive in-depth sequencing to identify naturally occurring genetic mutations that can be used to trace cell clones^13–16^. Although clonal tracking through natural mutations is a powerful technique that does not require any invasive maniputation^13–15,17–20^, it suffers from a prohibitive cost, low efficiency, and complications in clonal comparison that arise from the rarity and randomness of natural mutations. As an alternative to tracking natural mutations, some studies have employed synthetic DNA barcodes to simultaneously mark individual cancer cells^21–24^. Two recent studies have also demonstrated the feasibility of integrating synthetic DNA barcode tracking with single-cell mRNA sequencing analyses^25,26^. In conjunction with patient-derived xenograft (PDX) models, the use of synthetic DNA barcoding has greatly improved our understanding of the heterogeneous growth and metastasis of cancer cells^22,23,27,28^.

In this study, we present an integrated experimental system that directly connects gene expression with cellular behavior at the single-cell level by combining synthetic DNA barcode tracking and single-cell mRNA sequencing in a PDX model. We have previously demonstrated the high sensitivity and precise quantification of our DNA barcode tracking using hematopoietic stem cells in vivo^29–33^. Here, we adapt the barcode tracking to assay the activities of cancer cells and their gene expression profiles simultaneously in a PDX model xenografted by human B-cell acute lymphoblastic leukemia (B-ALL) samples. In the PDX model, B-ALL cells can autonomously home to their native micro-environment in the bone marrow and can be easily and repeatedly sampled over time to monitor cancer progression and therapeutic response. Using this integrated system, we show that primary B-ALL clones exhibit heterogeneous dynamics during expansion, circulation, and response to chemotherapy. Furthermore, their distinct temporal and spatial clonal dynamics are associated with unique gene expression.

## Results

### Integrating single-cell transcriptome and clonal tracking

**Fig. 1:**
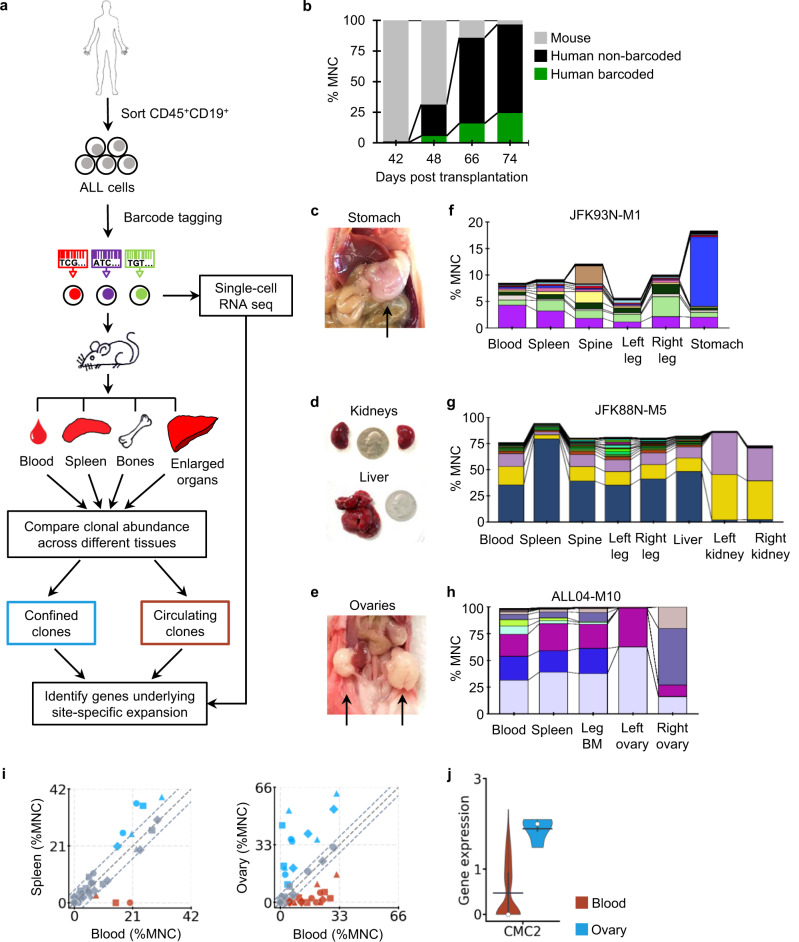
Distinct leukemia clones expanded in extramedullary organs. **a** Clonal abundance was assessed in different tissues and organs and mapped to single-cell RNA sequencing data. **b** The fractions of mouse cells, non-barcoded human leukemia cells (GFP−), and barcoded human leukemia cells (GFP+) in the mononuclear cells (MNC) of the peripheral blood from a xenografted mouse over time. **c**–**e** Representative images showing extramedullary sites of leukemia expansion. Quarters are shown in (**d**) as size references. Additional mice are shown in Supplementary Fig. 4. **f**–**h** Clonal distribution across different tissues and organs in representative mice. Each color represents one distinct genetic barcode corresponding to a leukemia clone. Additional mice are shown in Supplementary Figs. 5 and 6c, f. **i** Comparison of clonal abundance between the peripheral blood and the spleen or the ovary. Markers of the same shape represent data from one mouse. 99% confidence intervals of correlation were determined by the blood and spleen comparison and highlighted by dashed gray lines. **j** The *CMC2* gene was significantly upregulated in clones more abundant in the ovary than clones more abundant in the blood. The black bar indicates the mean, and the white dot represents the median. Source data are provided as a Source Data file.

Primary B-ALL cells were genetically barcoded using a GFP-encoding lentiviral vector (Fig. 1a and Supplementary Table 1)^29,30^. After barcode labeling, they were transplanted into sub-lethally irradiated NSG or NSG-SGM3 mice. Subsequent clonal tracking assays were performed as previously described^29,30^. Our data show that the barcoded leukemia cells expanded proportionally to non-barcoded leukemia cells in recipient mice (Fig. 1b and Supplementary Fig. 1), suggesting that barcoded cells are representative of the engrafted leukemic cell population in terms of their progression in mice. As DNA barcodes are inserted into cellular genomes, they are inherited by all descendants of barcoded cells, allowing us to track cellular proliferation and elimination. In this study, a “clone” refers to cells carrying identical barcodes.

To assay single-cell gene expression, a fraction of the donor barcoded cells was analyzed by droplet-based single-cell transcriptome analyses^34,35^ (Supplementary Fig. 2), while the rest of the donor barcoded cells were xenografted into mice to assay cellular activities (Fig. 1a). During the single-cell transcriptome assays, cDNAs from each cell were tagged by a unique DNA sequence that is used as a cellular index. Since the clonal tracking barcodes are transcribed, they can be recovered from the single-cell cDNA library together with the cellular indexes. The molecules containing both clonal tracking barcodes and cDNA cellular indexes were selectively amplified from the single-cell cDNA library and sequenced to attain efficient mapping between single-cell gene expression and the clonal activity that was examined in the subsequent recipients (Fig. 1a and Supplementary Fig. 3).

### Distinct leukemia clones expand in extramedullary organs

All recipient mice that received cells from three particular patient samples exhibited extramedullary expansion (Fig. 1c–e and Supplementary Fig. 4). Each of these mice exhibited an enlargement of a specific organ—enlarged kidney, stomach or ovaries—depending on the particular patient sample it received (Fig. 1c–e and Supplementary Fig. 4). We analyzed the human cells from these enlarged organs and found that leukemia clones at the extramedullary sites were often different from those in the hematopoietic tissues, including the peripheral blood (Fig. 1f–h, Supplementary Figs. 5 and 6c, f). This demonstrates the existence of clonal selection during extramedullary expansion. We analyzed the gene expression profiles of donor cells, comparing clones that expanded in the ovary with those that did not (Fig. 1i). We identified a largely unknown gene, *CMC2* (COX assembly mitochondrial protein 2 homolog), that was expressed at a significantly higher level in clones overrepresented in the ovary (Fig. 1i, j, and Supplementary Fig. 7). *CMC2* was identified as playing essential roles in regulating *Sod1* activity^36^, and *Sod1* is known to regulate the progression and metastasis of various types of cancers^37–39^. Our data revealed that the B-ALL clones that expanded in the ovary expressed elevated levels of *CMC2* prior to transplantation, suggesting that B-ALL cells that expand in the ovary had distinct gene expression prior to adapting to the ovary microenvironment.

### Spatially confined clonal expansion in the bone marrow

**Fig. 2:**
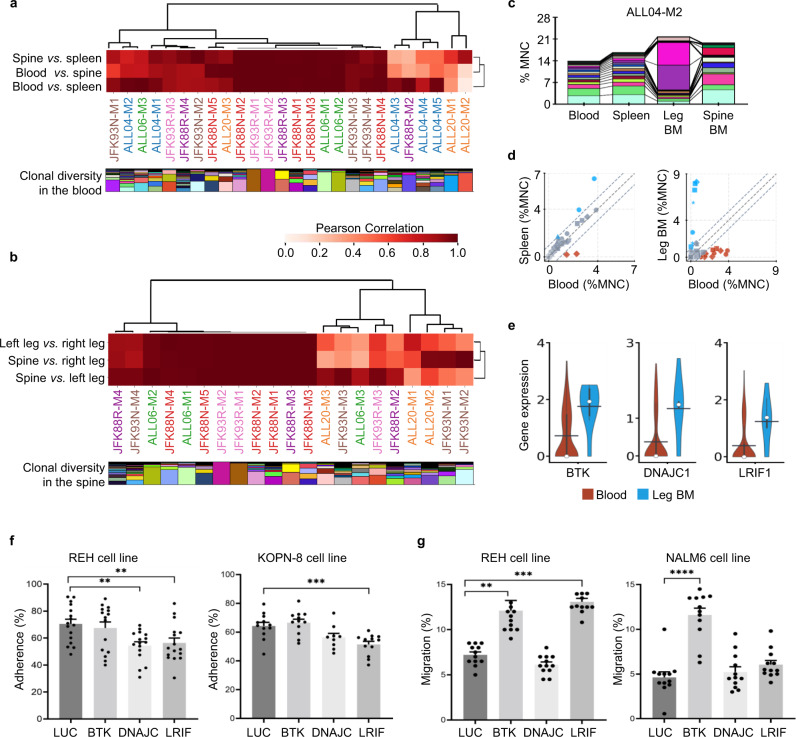
Spatially confined expansion of leukemia clones in the bone marrow of primary recipients. **a**, **b** Heatmaps showing Pearson Correlations of clonal abundance across different tissues and organs. Each column corresponds to one primary recipient mouse. The clonal diversity of each mouse is shown below, where each color represents a unique clone. **a** Comparison between the peripheral blood, spleen, and bone marrow from the spine. **b** Comparison of the bone marrow cells harvested from three distinct anatomical regions: left leg, right leg, and spine. **c** Leukemia clonal diversity across different tissues and organs from one representative mouse. Additional mice are shown in Supplementary Fig. 6. Each color represents one distinct genetic barcode corresponding to a leukemia clone. **d** Comparison of clonal abundance between the peripheral blood and the spleen or the leg bone marrow for ALL04 clones. Markers of the same shape represent data from one mouse. 99% confidence intervals were determined by the blood and spleen comparison and highlighted by dashed lines. **e** Genes significantly differentially expressed in ALL04 clones more abundant in the BM compared to clones more abundant in the blood. The black bar indicates the mean, and the white dot represents the median. **f**, **g** Genes identified in (**e**) and a negative control luciferase (*LUC*) gene were knocked out using the CRISPR/Cas9 technology in B-ALL cell lines (REH, KOPN-8, and NALM6). **f** Adhesion of the B-ALL cells to OP9 stroma cells was analyzed after 48 h of co-incubation. Four independent experiments for REH (*n* = 16 for *LUC*, *DNAJC*, *LRIF*; *n* = 15 for *BTK*). Three independent experiments for KOPN-8 (*n* = 12 for *LUC*, *BTK*, *LRIF*; *n* = 9 for *DNAJC*). **g** Migration of the B-ALL cells was analyzed after 12 or 24 h of incubation (12 h for NALM6, 24 h for REH). Three independent experiments (*n* = 12). **f**, **g** Data shown as mean ± SEM. ^**^P < 0.01, ^***^P < 0.001, by two-sided *t* test without adjustment. MNC mononuclear cells. BM bone marrow. Source data are provided as a Source Data file.

To determine the cellular heterogeneity of leukemia circulation, we compared the clonal composition between different tissues and organs in the primary recipient mice of seven B-ALL samples from five patients. We found that leukemia clones in the blood and spleen were highly correlated in all mice, except for two mice that received the same patient sample (ALL20-M1 and ALL20-M2). In these two mice, one clone dominated the blood, but this clone was not abundant in any other tissues (Fig. 2a and Supplementary Fig. 6a). In a few mice, clones in the bone marrow did not correlate well with those in the blood and the spleen (Fig. 2a). We then compared the bone marrow from different anatomical sites and found that more than half of the primary recipient mice exhibited high clonal correlation while others showed significant differences (Fig. 2b). Mice that received ALL20 and JFK93N samples mostly exhibited considerable differences in clonal correlation (Fig. 2b), suggesting that a cell-intrinsic mechanism is responsible for tissue homing patterns.

The clonal disparity of the leukemia cells in the bone marrow uncovered by our barcode clonal tracking challenges the use of a single biopsy during diagnosis which assumes that leukemia, a “liquid” cancer, uniformly spreads throughout the body (Fig. 2a, b)^40,41^. A recent study also showed asymmetrical clonal distribution across different bones and extramedullary sites^24^. Here, we discovered that the clonal discrepancy mainly arises from a small number of clones that substantially expand in the bone marrow at single anatomical sites and that do not circulate (Fig. 2c and Supplementary Fig. 6). This spatially confined clonal expansion was consistently detected in 15 primary recipient mice that received B-ALL cells from 5 different patients (Fig. 2c and Supplementary Fig. 6a–e). It was not detected in any mice that received one particular patient sample (JFK88N) (Supplementary Fig. 6f), suggesting that it is a cell-autonomous characteristic.

To identify the genes associated with the spatially confined clonal expansion in the bone marrow, we performed single-cell RNA sequencing analyses of the donor ALL04 cells. We compared the gene expression profiles of the clones that were more abundant in the bone marrow with those that were more abundant in the peripheral blood (Fig. 2d). We found three genes—*BTK, DNAJC*, and *LRIF1*—that were significantly differentially expressed (Fig. 2e and Supplementary Fig. 8). *BTK* (Bruton’s tyrosine kinase) is critical for signal transduction downstream of the pre-B cell receptor (*pre-BCR*) and functions as a tumor suppressor in B-ALL^42,43^. A *BTK*-binding molecule, Ibrutinib, is currently being tested in a clinical trial for treating B-ALL (ClinicalTrials.gov Identifier: NCT02997761). Our data here associate the expression of *BTK* with spatially confined clonal expansion (Fig. 2d, e). The other two genes that we identified, *DNAJC* and *LRIF1*, could also be potential therapeutic targets for future studies.

To test the functional roles of *BTK*, *DNAJC*, and *LRIF1* in spatially confined clonal expansion, we used CRISPR/Cas9 technology to knock out these genes in B-ALL cell lines. We found that these genes did not influence the growth of B-ALL cells (Supplementary Fig. 9). However, knockout of *DNAJC* or *LRIF1* significantly reduced the adherence of the B-ALL cells to the OP9 stroma cells, suggesting they may play a role in enhancing cell adherence to the bone marrow niche (Fig. 2f). In addition, knockout of *BTK* or *LRIF1* significantly increased the migration of the B-ALL cells, suggesting their potential involvement in regulating homing to the bone marrow environment (Fig. 2g). The data from B-ALL cell lines suggest that the increased expression of *BTK, DNAJC*, and *LRIF1* in the clones expanded in the bone marrow may have promoted the observed clonal expansion by regulating the homing and adherence of B-ALL cells to the bone marrow niche (Fig. 2e). Consistent with our finding, a phase II study of chronic lymphocytic leukemia reported that the BTK-binding molecule, Ibrutinib, caused an efflux of tumor cells from the tissue compartments into the blood^44^.

### Clonal selection occurs during serial transplantation

**Fig. 3:**
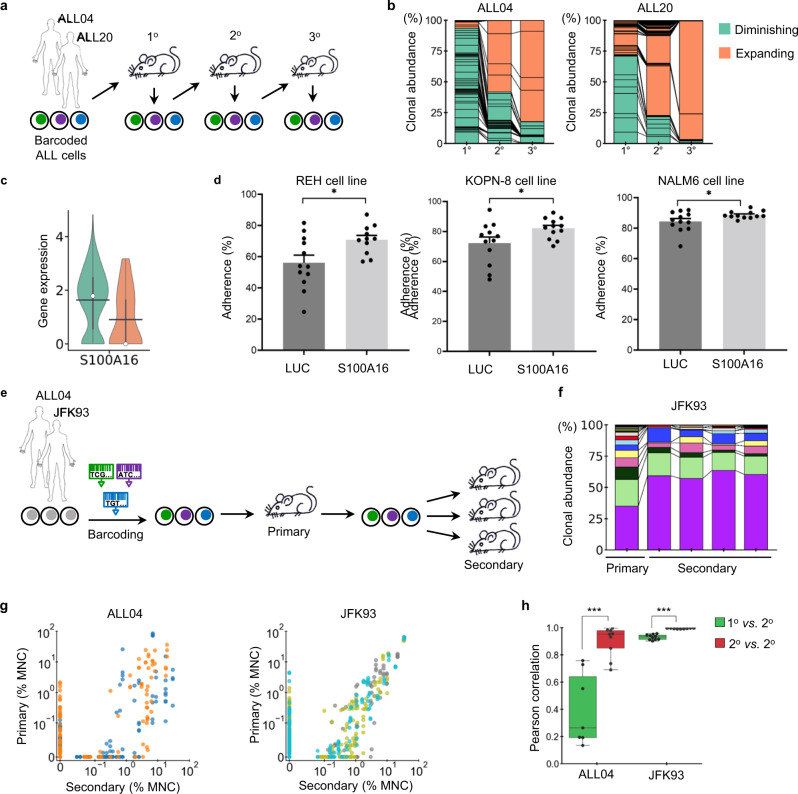
Clonal selection occurs during serial transplantation. **a** Barcoded B-ALL cells were consecutively transplanted through three generations of mice. Clonal abundance was assessed in each recipient. **b** K-means clustering separated leukemia clones into diminishing and expanding groups. Each section in the column represents the average abundance of each clone from different mice. **c** Gene *S100A16* was significantly differentially expressed between the diminishing and expanding ALL04 clones as classified in (**b**). Black bar indicates the mean, and white dot represents the median. **d** *S100A16* and a negative control luciferase (*LUC*) gene were knocked out using the CRISPR/Cas9 technology in B-ALL cell lines (REH, KOPN-8, and NALM6). Adhesion of the B-ALL cells to OP9 stroma cells was analyzed after 48 h of co-incubation. Three independent experiments (*n* = 11 for REH; *n* = 12 for KOPN-8 and NALM6). Data are shown as mean ± SEM. ^*^P < 0.05, by two-sided *t* test without adjustment. **e** Human B-ALL cells were barcoded before primary transplantation and transplanted into multiple secondary recipients. **f** Clonal abundance changes during serial transplantation. Each color represents a genetic barcode corresponding to a leukemia clone. Each column shows data from one mouse. Additional mice are shown in Supplementary Fig. 14. **g** Clonal abundance comparison between the primary and secondary recipients during serial transplantation. Each dot represents a barcoded clone, and each color depicts one primary mouse. MNC mononuclear cells. **h** Pearson correlation of clonal abundance between different mice during serial transplantation. ^***^P < 0.001. Each dot represents a comparison between a pair of mice. Black lines in the boxes denote median values. Boxes extend from the 25th to the 75th percentile of each group’s distribution of values. *P* values were calculated by the two-sided *t* test without adjustment. All clonal abundance data in Fig. 3 originates from spleen cell analyses. Barcoded spleen cells were transplanted during serial transplantation. Source data are provided as a Source Data file.

The spatially confined clonal expansion was missing in secondary recipient mice (Supplementary Fig. 5), because they are under-represented in the spleen where donor cells for consecutive transplantation are collected by common convention. This explains why the spatially confined clonal expansion was not identified by previous studies as most PDX studies use mice after multiple passages in order to obtain enough biological replicates from limited patient samples. To determine how clonal diversity is influenced by transplantation, we performed serial transplantations of barcoded leukemia clones recovered from the spleen and compared the clonality of spleen cells between different recipients (Fig. 3a). Comparisons of the primary, secondary and tertiary recipients show that clonal abundances generally change in a monotonic way with passages, where clonal abundances either constantly diminish or constantly expand (Fig. 3b).

We compared the gene expression profiles between the diminishing clones and expanding clones using the single-cell RNA sequencing analyses of the donor ALL04 cells. We found one gene, *S100A16*, that was significantly differentially expressed (Fig. 3c and Supplementary Fig. 10). *S100A16* is a member of the S100 calcium-binding protein family and has been associated with proliferation, migration, and invasion of multiple types of cancers^45–48^. A recent study of Ph-negative B-cell ALL patients showed that *S100A16* suppresses the growth and survival of B-ALL cells, consistent with our finding that its expression is significantly higher in diminishing clones^48^ (Fig. 3c). CRISPR/Cas9 knockout of this gene (Supplementary Fig. 11) increased the adherence of B-ALL cells to OP9 stroma cells in all three cell lines that were tested and increased the migration in one of the three B-ALL cell lines, without changing their growth rates (Fig. 3d and Supplementary Figs. 12 and 13). The data from B-ALL cell lines suggest that the reduced expression of *S100A16* in the expanding clones during serial transplantation may play a role in promoting the engraftment of B-ALL cells (Fig. 3c).

Comparing multiple secondary recipient mice that received cells recovered from the same primary recipient (Fig. 3e), we found that some clones abundant in primary recipient mice were not detected in secondary recipients (Fig. 3f, g). The clonal correlations between secondary recipients were significantly elevated, suggesting that clonal selection is cell-autonomous (Fig. 3f, h and Supplementary Fig. 14). The consistent clonality across different secondary recipients allows different therapeutic treatments to be tested on the same set of clones in different mice.

### Recapitulating B-ALL combination therapy using PDX model

The efficacy of chemotherapy in treating ALL is one of the great successes in medical oncology—transforming a universally fatal disease into a curable one for most children and many adults. One peculiarity of ALL therapy is that multiple cycles of low-dose maintenance therapy after high-intensity therapy are necessary for long-term cure (NCCN clinical practice guidelines: Acute Lymphoblastic Leukemia Version 2.2019; Pediatric Acute Lymphoblastic Leukemia Version 1.2020). This complex regimen was derived empirically from decades of methodical clinical research. However, a mechanistic explanation of how intensive and maintenance therapies synergize has never been presented. We hypothesized that intensive and maintenance therapies target different subsets of ALL clones. To test this hypothesis, we transplanted identical B-ALL clones into multiple mice that were subsequently treated with different types of chemotherapy. While various chemotherapeutic agents have been used in B-ALL treatment, we used a multi-agent therapy consisting of vincristine, dexamethasone, and l-asparaginase for the intensive phase^49,50^ and a single-agent therapy consisting of methotrexate for the maintenance phase, mimicking clinical practice. Experimental mice were randomly assigned to five groups: (i) vehicle control; (ii) combination therapy consisting of short-term intensive therapy followed by prolonged maintenance therapy, designed to approximate the clinical ALL treatment; (iii) short-term intensive therapy; (iv) prolonged intensive therapy; and (v) prolonged maintenance therapy. Maintenance therapy was applied weekly at a low dose throughout the lives of the mice. Prolonged intensive therapy was applied to the toleration of the mice as assessed by changes to their body weight (Supplementary Fig. 15).

**Fig. 4:**
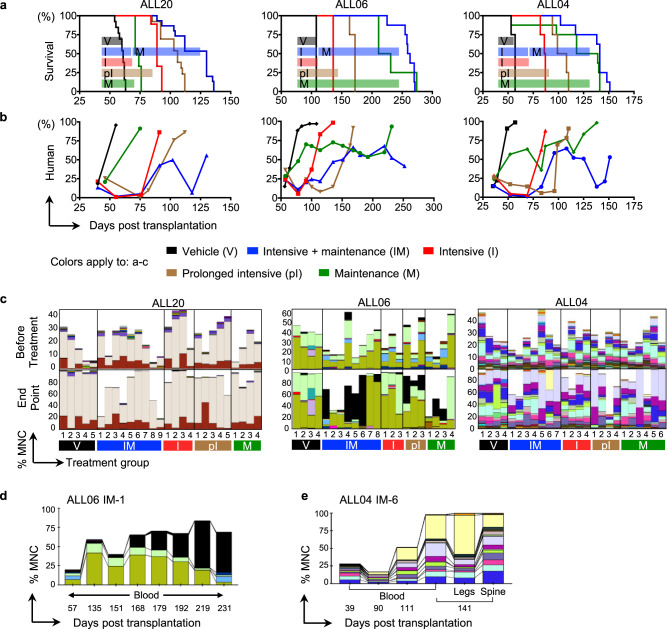
Comparing the same leukemia clones during different chemotherapy treatments across multiple mice. **a** Kaplan–Meier survival plots of PDX mice under various treatments. Color bars illustrate the duration of the treatment for each group. **b** Human chimerism in the peripheral blood. Shown are the means of all experimental mice. Mouse variations are provided in Supplementary Fig. 16. **c** Clonal abundance in mouse blood before treatments and at the end time point for each mouse. Each column represents one mouse. Each color represents one distinct genetic barcode corresponding to a leukemia clone. **d**, **e** Representative clonal dynamics during combination therapy in two PDX mice. Additional mice are shown in Supplementary Figs. 17–19. The black-colored clone in multiple mice of (**c**, **d**) and Supplementary Fig. 18 represents non-barcoded (GFP−) human cells from patient ALL06. PCR analysis reveals that these GFP− human cells did not carry any genetic barcode (Supplementary Fig. 20). They may have originated from a clone that had lost the barcode construct or that may have been collected due to a sorting error. These non-barcoded human cells were not detected in mice that received the vehicle treatment or short intensive therapy, or in any mice that received sorted GFP+ B-ALL cells derived from the other patient samples. Source data are provided as a Source Data file.

**Fig. 5:**
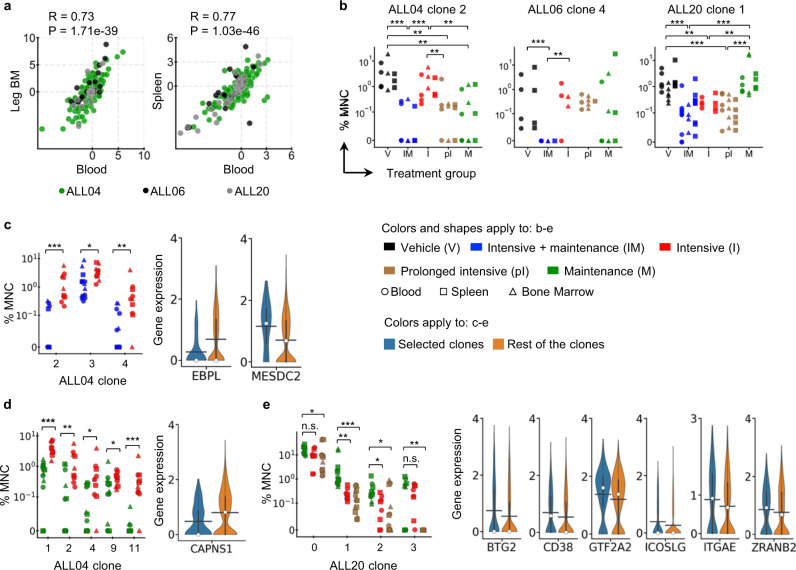
Some B-ALL clones respond differentially to intensive and maintenance chemotherapy in distinct but consistent manners across multiple mice. **a** Comparing chemotherapy response of the same clones in different tissues. Shown are the log_2_ fold differences between the average clonal abundances of two chemotherapy treatments for each tissue. R Pearson’s correlation coefficient, *P* two-tailed *P* value. Each dot represents one clone. **b** Example clones that responded differently under various chemotherapy treatments. Each marker represents data from one tissue and one mouse. **c** Left, ALL04 clones that responded better to combination therapy than to intensive therapy. **d** Left, ALL04 clones that responded better to maintenance therapy than to intensive therapy. **e** Left, ALL20 clones that responded better to intensive therapy than to maintenance therapy. **b**–**e** *P* values were calculated by a two-sided Kruskal Wallace test with Bonferroni correction. **c**–**e** Right, genes significantly differentially expressed in the clones shown on the left (blue) compared to all other clones from the same patient samples (orange). The black bar indicates the mean, and the white dot represents the median. BM bone marrow, MNC mononuclear cells. *P* values were calculated by the one-sided Mann–Whitney *U*-test and adjusted using both the experimental data and the scramble data, see the Methods section for detail. Source data are provided as a Source Data file.

We tested these five regimens on 74 mice that received B-ALL cells from three patients (Figs. 4 and 5). As expected, mice that received chemotherapy survived longer than those that did not (Fig. 4a). Mice that received the combination therapy consistently survived the longest (Fig. 4a). The survival rate was directly correlated with the fraction of human leukemia cells in the mouse peripheral blood (Fig. 4b and Supplementary Fig. 16). Furthermore, intensive therapy effectively and rapidly removed the vast majority of leukemia cells, while maintenance therapy suppressed leukemia growth (Fig. 4b and Supplementary Fig. 16). Mouse body weights dropped quickly in the absence of chemotherapy due to the leukemia burden (Supplementary Fig. 15). The bodyweight drop was also evident following intensive therapy (Supplementary Fig. 15), consistent with the toxicity of intensive treatment observed in clinical practice. Taken together, our experimental model well recapitulates the key aspects of the two phases of ALL therapy.

### Distinct chemotherapy responses of B-ALL clones

We compared leukemia clones before and after various treatments and noted the emergence of previously undetected clones after chemotherapy treatment (Fig. 4c). The emergent clones were abundant at several later time points in multiple tissues (Fig. 4d, e, and Supplementary Figs. 17–20). Their emergence indicates their survival and growth advantages over other clones after chemotherapy. Since mice receiving the same patient sample exhibited similar clonal compositions prior to chemotherapy (Fig. 4c), we were able to determine the effects of different treatments by comparing the clonal composition of mice after the treatments. Identical clones in the peripheral blood, spleen, and bone marrow exhibited consistent chemotherapeutic responses (Fig. 5a). Moreover, some clones exhibited significant consistency in their chemotherapy responses across different mice (Fig. 5b–e and Supplementary Figs. 21–24). For example, ALL04 clone 2 consistently responded well to combination therapy, prolonged intensive therapy, and maintenance therapy, but not to short-term intensive therapy. ALL06 clone 4 only responded to combination therapy in all mice. ALL20 clone 1 did not respond to maintenance therapy in any mice but consistently responded to all treatments that involved an intensive therapy phase (i.e., combination therapy, short-term, and prolonged intensive therapy).

While no clone was found to respond better to intensive therapy than to combination therapy (Supplementary Figs. 21–24), several clones from ALL04 and ALL06 responded significantly better to combination therapy than to intensive therapy alone (Fig. 5c and Supplementary Fig. 24). We performed single-cell RNA sequencing analysis on donor B-ALL cells to determine if the clones that exhibited a distinct chemotherapy response share a common gene expression characteristic prior to treatment. In ALL04, the clones that responded better to combination therapy than to intensive therapy expressed significantly lower levels of *EBPL* and significantly higher levels of *MESDC* compared to other clones (Fig. 5c and Supplementary Fig. 25). In addition, some clones from ALL04 responded significantly better to maintenance therapy than to intensive therapy. Compared to other clones, these clones expressed lower levels of *CAPNS1* prior to chemotherapy (Fig. 5d and Supplementary Fig. 26). This finding is in line with a previous study showing that inhibiting *CAPNS1* sensitizes prostate cancer cells to methotrexate treatment^51^.

In another patient sample ALL20, some clones responded significantly better to intensive therapy than to maintenance therapy (Fig. 5e). Prior to chemotherapy treatments, these clones expressed higher levels of *BTG2*, *CD38*, *GTF2A2*, *ICOSLG*, *ITGAE*, and *ZRANB2* compared to other clones (Fig. 5e and Supplementary Fig. 27). *BTG2* is a tumor suppressor in B-ALL and a known target of *p53*^52^. It is upregulated during chemotherapy-mediated apoptosis in cancer cells^53,54^. Our data suggest that the upregulation of *BTG2* sensitizes B-ALL clones to intensive chemotherapy treatment. *CD38* and *ITGAE* (*CD103*) are both activation markers of leukemia, and our data is consistent with the idea that intensive chemotherapy selectively targets the subset of cancer cells that are highly proliferative^55^. Monoclonal antibodies targeting *CD38* (daratumumab, isatuximab, and MOR202) have been used in many clinical trials for hematopoietic malignancies^56^. *CD38* and *ITGAE* were activated in response to pentostatin^55^, an antimetabolite drug that disrupts nucleic acid synthesis like methotrexate. This is consistent with our finding that clones with higher expression of these genes were less sensitive to methotrexate. *ICOSLG* has been found to be upregulated in trastuzumab-resistant breast cancer cells^57^, suggesting that it plays a role in therapeutic resistance. Taken together, the data provide original experimental evidence that different subsets of ALL clones are significantly different in their response to intensive and maintenance therapies and possess distinct gene expression prior to chemotherapy.

### Relapsed B-ALL samples exhibit increased clonal dominance

**Fig. 6:**
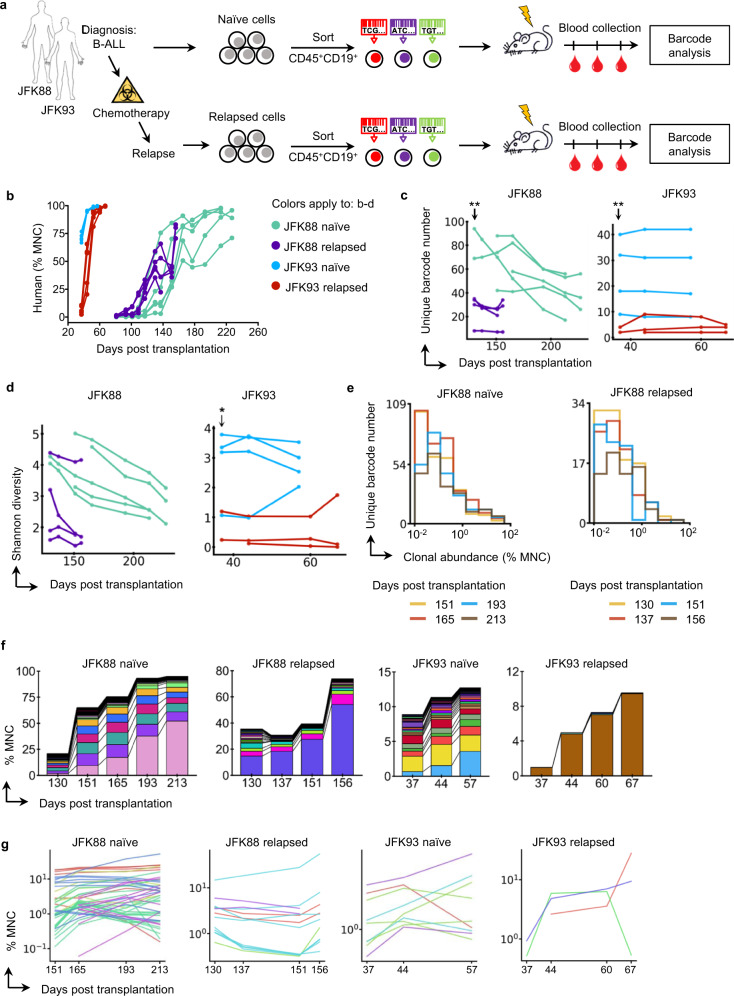
Relapsed leukemia cells generate greater clonal dominance in the PDX model than chemotherapy-naïve cells from the same patients. **a** Human B-ALL cells acquired from two patients at distinct disease stages (chemotherapy naïve and chemotherapy relapsed) were genetically labeled with DNA barcodes and transplanted into irradiated immunocompromised mice. Leukemia progression was monitored through peripheral blood analysis. **b** Human chimerism of the mouse peripheral blood following transplantation of diagnostic (naïve) and relapsed leukemia cells from the same patients. Each line depicts data from one mouse. **c** Number of unique DNA barcodes detected in the peripheral blood. Each line depicts data from one mouse. **P < 0.01. **d** Clonal diversity in the peripheral blood over time. Each line represents data from one recipient mouse. *P < 0.05. **c**, **d** Independent two-sided *t* test without adjustment. **e** Clonal abundance distribution changes over time. The histogram shows data from all mice. **f** Clonal composition in the peripheral blood of mice xenografted by naïve and relapsed samples of the same patient. Each color represents one distinct genetic barcode corresponding to a leukemia clone. Additional mice are shown in Supplementary Fig. 31. **g** Clonal abundance change over time. Each line represents one clone. Each color depicts one mouse. Shown are all clones exceeding 1% of mononuclear cells (MNC) abundance at any time point. Source data are provided as a Source Data file.

To directly compare primary B-ALL cells before and after chemotherapy treatment in patients, we collected human B-ALL samples from the diagnosis (naïve) stage and after relapse from each of the two patients (Fig. 6a and Supplementary Table 1). The samples from patient JFK93 grew much faster than those from patient JFK88 in vivo (Fig. 6b) and in vitro (Supplementary Fig. 28). Our barcode analysis showed that less than 1% of donor cells engrafted (Supplementary Fig. 29), highlighting the severe engraftment barrier of the PDX model. In addition, significantly more barcodes were detected in the peripheral blood of mice xenografted with naïve samples than those xenografted with relapsed samples (Fig. 6c, for both JFK88 and JFK93: *P* value < 0.01 at the initial time points; *P* value < 0.001 across all time points). The clonal diversity was also much higher for the naïve samples compared to the relapsed samples from both patients (Fig. 6d), although barcode transduction was abnormally high in one naïve sample under the same experimental condition (Supplementary Fig. 30).

After transplantation, the number of leukemia clones derived from the slow-growing samples of patient JFK88 generally decreased over time (Fig. 6c). This reduction was mainly caused by the elimination of low abundance clones (Fig. 6e, f and Supplementary Fig. 31). While the growth of high abundance clones was generally stable (Fig. 6f and Supplementary Fig. 31), the clonal competition was evident during the later disease stages when the overall growth slowed down (Fig. 6b, g). In mice that received the JFK93 naïve sample, exactly one clone outcompeted other clones over time (Fig. 6g). Most mice that received relapse samples were dominated by just one or two clones through all the time points that we examined (Fig. 6f and Supplementary Fig. 31), suggesting that cells in the relapse samples are more heterogeneous than those in the naïve samples in terms of their engraftment and expansion efficiency in PDX mice. The data from both patients indicate that intratumoral heterogeneity may be more prominent during the relapse phase compared to the naïve phase and that PDX models using patient samples derived during relapse may be severely prejudiced.

## Discussion

In this study, we directly linked the intratumoral clonal dynamics of individual leukemia cells with their heterogeneous gene expression. We showed that individual leukemia cells exhibit different levels of expansion (Figs. 3 and 6), circulation (Figs. 1 and 2), and chemotherapy response (Figs. 4 and 5). We identified the genes associated with these cellular activities by comparing cells within the same patient samples that exhibited different levels of cellular activity (Figs. 1, 2, and 5). Our clustering analyses using single-cell transcriptome data were not able to identify any cluster that was significantly correlated with the clonal behaviors examined in this study (Supplementary Fig. 2). It is possible that clonal differences also reside at the epigenetic level^58^. Only a few genes were significantly differentially transcribed between clones exhibiting distinct behaviors (Figs. 1, 2, and 5). Among these genes, approximately half of the identified genes have been previously associated with similar roles in cancer biology (e.g., *BTK*, *CAPNS1*, *BTG2*, *CD38*, *ICOSLG*, and *ITGAE*)^42,43,51–57^. In addition, we identified genes of previously unknown cancer relevance with regards to leukemia circulation (e.g., *LRIF1*, *DNAJC1*, and *CMC2*) and therapeutic treatment (e.g., *EBPL*, *MESDC2*, *ZRANB2*, *GTF2A2*) (Figs. 1, 2, and 5). These genes could be promising therapeutic targets. These genes were identified using a small number of patient samples and validated using cell lines in vitro (Figs. 1, 2, and 5). Future studies using more patient samples and in vivo tests can further validate their translational potentials. Furthermore, our methodology—identifying genes by assaying intratumoral heterogeneity—can readily be applied to other diseases and biological processes.

Our findings highlight the inherent low engraftment problem of the PDX model by showing that less than 1% of donor cells engraft (Supplementary Fig. 29). The engraftment efficiency reflects how well the patient cells adapt to the microenvironment in mice. It is unclear if the engrafted cells in the PDX mice are representative of the entire patient cell population in each assay as this cannot be tested by any existing experimental model. In addition, our PDX model, as in most xenograft studies, uses immune-deficient mice to allow the engraftment of patient samples. The absence of the immune system limits direct translation to the clinical condition. The PDX model is the primary animal model used in cancer studies and drug discovery^27,28,59–62^. It is also regularly used as the final assay prior to clinical trials in humans^63–65^. Here, we show that the clonal diversity was worse in PDX mice of late passages (Fig. 3), which are commonly used in therapeutic development^59,66–68^. Furthermore, when xenografted with two patient samples collected after relapse, most PDX mice became dominated by cells that descended from a single patient cell (Fig. 6). As PDX studies generally do not discriminate between samples collected from different disease stages, our data demonstrate that certain samples may be severely biased. The sampling limitations of the PDX model are particularly troubling in light of the heterogeneity of cancer cells^1–3,5,6^.

Our data precisely delineate the heterogeneous clonal dynamics of leukemia using robust biological replicate assays that eliminate the influence of rare molecular events, such as random viral integration^36–40^. Using 5 primary patient samples and 15 primary xenograft mice, we showed that dominant clonal expansion in the bone marrow is commonly restricted to confined anatomical sites (Fig. 2 and Supplementary Fig. 6). This surprising finding suggests that bone marrow biopsies, widely used for diagnosis^40,41^, may not accurately represent the overall disease condition. Spatially confined leukemia clones expand aggressively without circulating (Fig. 2 and Supplementary Fig. 6). As they are not collected for subsequent transplantation, they were missed by prior studies that relied on xenografts of later passages. In addition, we provide a direct mechanistic explanation for the famed success of combination therapy in ALL treatment^69,70^. We show that some ALL clones exhibit consistent and distinct responses to intensive and maintenance chemotherapy across different mice in our chemotherapy treatment model (Figs. 4 and 5), supporting the hypothesis that intensive and maintenance therapies synergize by targeting different subsets of ALL clones. Future experiments using other chemotherapeutic agents and treatment conditions may provide further insights. Our study can help improve treatments for other types of cancer by offering a strategy to identify and characterize treatment-resistant cells through deciphering intratumoral heterogeneity.

## Methods

### Human cells

Clinical specimens were obtained from adult patients with B-ALL (Supplementary Table 1). All human subjects provided informed consent, and the study was approved by the University of Southern California institutional review board. Mononuclear cells (MNC) were isolated by density centrifugation using Ficoll Paque Plus, density 1.077 (GE Healthcare Bio-Sciences) followed by two washes with Iscove’s Modified Dulbecco’s Medium (IMDM) (Thermo Fisher Scientific) and were frozen for later uses. During recovery, frozen cells were thawed and cultured in IMDM supplemented with 20% fetal bovine serum (FBS) (VWR Life Science Seradigm) for 1–2 h at 37 °C. Cells were then stained, analyzed, and sorted. All patients provided written consent. Ethics oversight was provided by ECOG-ACRIN and CTEP through CTEP protocol E2993T5 and by the USC Health Sciences Institutional Review Board.

### Leukemia cell culture and lentiviral transduction

Human B-ALL cells were sorted for human CD45 and CD19 from cryopreserved samples (Supplementary Table 2). These cells were either primary human bone marrow aspirates (ALL04, ALL06, and ALL20) or passaged in mice and recovered from the spleen (JFK88 and JFK93). Cells were cultured in StemSpan^TM^ Serum-Free Expansion Medium II (SFEM II) (Stem Cell Technologies) in the presence of 20 ng/ml human FLT-3 ligand, 20 ng/ml human Interleukin-3 (IL-3), and 50 ng/ml human Stem Cell Factor (SCF) (all from Gibco by Life Technologies). After 24 h of pre-stimulation under the culture condition, cells were washed and incubated for another 16 h in the same medium with the addition of lentivirus carrying the DNA barcodes. Totally, 8 ng/µl polybrene was added to the culture to facilitate viral transduction. B-ALL cells were washed three times with Dulbecco’s Phosphate Buffered Saline (D-PBS) (Gibco by Life Technologies) prior to transplantation. The transduction rate was generally below 30% in order to ensure that each cell was marked by a single barcode.

### Mice

NOD.Cg-*Prkdc*^*scid*^ *Il2rg*^*tm1Wjl*^ (NSG, stock number 05557) and NOD.Cg-*Prkdc*^*scid*^ *Il2rg*^*tm1Wjl*^ Tg (CMV-IL3,CSF2,KITLG)1Eav/MloySzJ (NSG-SGM3, stock number 013062) mice were obtained from Jackson Laboratory. Mice were bred and maintained at the Research Animal Facility of the University of Southern California in a 12:12-h light–dark cycle, at room temperature (22 °C ± 1 °C) and humidity of 55% ± 10. Animal procedures were approved by the Institutional Animal Care and Use Committee of the University of Southern California. We have complied with all relevant ethical regulations.

### Human B-ALL engraftment

NSG or NSG-SGM3 mice were irradiated with 150 cGy and transplanted with 100,000–200,000 human B-ALL cells via tail vein injection. Mice were monitored daily for evidence of distress and were euthanized when human chimerism exceeded 90% of total MNC. At the end time point of each experiment, mouse peripheral blood was collected via perfusion using D-PBS with 10 mM ethylenediaminetetraacetic acid (EDTA) (Sigma-Aldrich). Spleen, bones, and tissues with noticeable extramedullary expansion were collected. Single-cell suspensions were prepared by crushing the tissues in D-PBS with 2% FBS and filtered through a 70 µM cell strainer. Cells were analyzed by flow cytometry. Totally, 500,000 barcoded cells were sorted for barcode analysis. Additional unsorted cells were frozen in IMDM with 20% FBS and 10% dimethylsulfoxide (DMSO) (Sigma-Aldrich) for later use.

### Blood sample collection and FACS analysis

Blood samples were collected into D-PBS containing 10 mM EDTA via a small transverse cut in the tail vein. A 2% dextran (Pharmacosmos) was added to remove red blood cells. To further eliminate red blood cells, the remaining blood cells were treated with ammonium-chloride-potassium lysis buffer on ice for 5 min. After a 45–60-min antibody incubation on ice, samples were suspended in D-PBS with 2% FBS and 4,6-Diamidino-2-phenylindole to distinguish dead cells. Cells were analyzed and sorted using the FACS-Aria cell sorters. Antibodies were obtained from eBioscience (currently Life Technologies/Thermo Fisher) and BioLegend (Supplementary Table 2). Flow cytometry data were analyzed using Diva software 8.0.1 (BD Biosciences).

### Chemotherapy treatment

Barcoded human B-ALL cells were transplanted into sublethally irradiated NSG or NSG-SGM3 mice. Once the human leukemia cell contribution reached 20–40% of total MNCs, mice were randomized and placed into one of the five chemotherapy groups: (i) vehicle control, (ii) combination therapy consisting of short-term intensive therapy followed by long-term maintenance therapy, (iii) short-term intensive therapy, (iv) prolonged intensive therapy, and (v) maintenance therapy. The intensive therapy consisted of Vincristine (Hospira Pharmaceuticals) (0.25 mg/kg) administered weekly via intravenous (IV) injection, Dexamethasone (AuroMedics Pharma) (7.5 mg/kg) administered Monday, Wednesday, and Friday via intraperitoneal (IP) injection, and l-asparaginase (Sigma-Tau Pharmaceuticals) (100 IU/kg) administered bi-weekly via IP injection. Maintenance therapy consisted of weekly Methotrexate (Accord Healthcare) (5 mg/kg) administered via IV or intramuscular injection.

The vehicle group received weekly IV injections of Bacteriostatic Water (Hospira Pharmaceuticals) during the life span of the mice. The short-term intensive therapy group only received four weeks of intensive therapy. The combination therapy group received four weeks of intensive therapy followed by maintenance therapy. The prolonged intensive therapy group was treated with intensive therapy to the toleration of the mice as assessed by changes to their body weight (Supplementary Fig. 15). Toxicity was assessed based on body weight change. The range of prolonged intensive therapy treatment was 7 weeks for ALL04 and ALL20, and 10 weeks for ALL06. The maintenance therapy group was treated with maintenance therapy continuously during the life span of the mice. The range of maintenance therapy treatment was 5 weeks for ALL20, 14 weeks for ALL04, and 28 weeks for ALL06.

Leukemia progression was monitored throughout the duration of treatment by analyzing the peripheral blood. In addition, mouse weight was monitored weekly throughout treatment to assess therapy toxicity. When bodyweight dropped more than 20% of the starting weight, chemotherapy doses were adjusted. Animal care was in accordance with institutional guidelines. Survival (Kaplan–Meier) curves were generated using GraphPad Prism Software (San Diego, California) with significance determined by the log-rank test.

### In vitro culture of B-ALL cell lines

B-ALL cell lines were maintained in RPMI (Gibco) supplemented with 10% FBS, penicillin/streptomycin, and GlutaMAX (Gibco) at 37 °C under humidified 5% CO_2_ condition. REH cells were a gift from the Parekh lab at Children’s Hospital Los Angeles (CHLA). Nalm6 cells were a gift from the Kim lab at CHLA. KPON-8 cells were a gift from the Chen lab at City of Hope. REH cells can be purchased from ATCC (cat. no. CRL-8286, RRID:CVCL_1650). Nalm6 cells can be purchased from ATCC (cat. no. CRL-3273, RRID:CVCL_UJ05). KOPN-8 cells can be purchased from DSMZ (cat. no. ACC-552, RRID:CVCL_1866).

### CRISPR/Cas9

Single guide RNAs (sgRNAs) targeting *BTK, LRIF, DNAJC*, and *S100A16* were designed using the Broad Institute CRISPick online tool. The sgRNAs were cloned into the BsmbI site of the pLKO5.sgRNA.EFS.tRFP lentiviral backbone (Addgene #57823). Five sgRNAs per gene target were cloned to overcome the limitations of the CRISPick algorithm. Correct insertion of sgRNA was verified by Sanger sequencing. Lentiviruses carrying the plasmid coding for *Cas9-GFP* or sgRNA-*tRFP* were generated in HEK293T cells (ATCC, cat. no. CRL-3216, RRID: CVCL_0063) by co-transfection of the packaging plasmids psPAX.2 and pCMV-VSV-G using BioT reagent (Bioland Scientific) following manufacturers’ protocols. sgRNAs targeting the same gene were mixed prior to lentiviral packaging to generate a single lentiviral pool per gene target. B-ALL cell lines were transduced in X-Vivo 15 media (Lonza, Walkersville Inc.) containing 10ug/mL polybrene. Transduced Cas9 cells were sorted using GFP on a BD S6 flow cytometer. Cas9-carrying B-ALL cell lines were then transduced with sgRNAs and sorted for double-positive GFP/tRFP on a BD S6 flow cytometer.

### T7 endonuclease 1 (T7E1) assay

To confirm CRISPR/Cas9 induced gene editing, B-ALL cells double transduced with Cas9-GFP and sgRNA-tRFP constructs were PCR amplified using primers for the sgRNA target regions under the following denaturation and reannealing conditions: 95 °C for 10 min, ramp down to 85 °C at 2 °C/s, ramp down to 25 °C at 0.1 °C/s. T7E1 digestion (NEB, Ipswich, USA) was performed at 37 °C for 20 min. The digested fragments were analyzed on a 2% agarose gel (Supplementary Fig. 11).

### Adhesion assay

Cas9 and sgRNA double transduced human B-ALL cells were cocultured with irradiated (5,000 cGy) OP9 mouse bone marrow stroma cells (ATCC) for 48 h at 37 °C under humidified 5% CO_2_ condition. Totally, 10,000 B-ALL cells were plated per well in a 96-well flat-bottom plate. Cells in the supernatant were collected after 48 h of coculture. Residual suspension cells were collected with one wash using PBS. Adherent cells were then detached using 0.25% trypsin EDTA solution (Gibco) and gentle pipetting. The suspension and adherent B-ALL cells were analyzed and quantified by FACS using human CD19 antibody (eBioscience, clone SJ25C1). The percent of adherence was calculated as the number of live hCD19+ adherent cells divided by the total number of live hCD19+ adherent cells and live hCD19+ suspension cells.

### Migration assay

Cells were cultured in 0.5% FBS for 12 h before seeding on an uncoated polycarbonate membrane insert (8.0-μm pores, Corning) in 24-well plates (Corning) at 5 × 10^4^ cells per well. Totally, 600 μl medium with 10% or 15% FBS was added to the lower compartment to form a chemoattractant gradient. After incubation at 37 °C for 12 h for NALM6 and 24 h for REH, the inserts were removed and cells that had migrated to the bottom were counted using a hemocytometer.

### DNA barcode analysis

The lentiviral DNA barcode construct and its application have been described in detail previously^30^. The DNA barcode was inserted into the 3′UTR region of *GFP* and transcribed together with *GFP*. Genomic DNA was extracted from sorted barcoded leukemia cells and amplified using a Phusion PCR master mix (Thermo Scientific, Waltham, MA). The PCR reactions were halted once they had progressed halfway through the exponential phase. PCR product was purified and analyzed using high-throughput sequencing. We combined sequencing data with FACS data to calculate the clonal abundance for each clone as indicated below. Clones with a clonal abundance greater than 0.01% were used for further analyses.

$${{Clonal}}\,{{abundance}}\, \%\ =\ 100\ast \left[\frac{\#\,{{of}}\,{{reads}}\,{{for}}\,{{each}}\,{{barcode}}}{{{total}}\,{{reads}}\,{{for}}\,{{all}}\,{{barcodes}}}\right]\left[\frac{\#\,{{of}}\,{{human}}\,{{cells}}}{{{total}}\,{{MNC}}}\right]\left[\frac{\#\,{{of}}\,{{GFP}}\,{{cells}}}{{{total}}\,{{human}}\,{{cells}}}\right]$$

### Single-cell RNA sequencing and data analysis

Single-cell RNA sequencing (scRNA-seq) was performed following the manufacturer’s protocol for the Chromium Single Cell 3’ Library (10× Genomics, V2) with minor modifications as follows. Barcoded ALL04 and ALL20 cells (hCD45+ GFP+) were sorted and loaded into the Chromium chip. After cDNA amplification, half of the amplified cDNA was used for the downstream fragmentation, adapter ligation, and sample index PCR. The other half of the amplified cDNA was used to PCR amplify molecules that contain both the genetic tracking barcodes and the Chromium cellular barcodes. These molecules were then sequenced using the SMRT sequencing platform (Sequel II, Pacific Biosciences) to map genetic tracking barcodes to Chromium cellular barcodes, and thereby connecting clonal tracking data and gene expression data from the same cell. The cDNA libraries were sequenced using an Illumina HiSeq 4000 at coverage of 50,000 raw reads per cell (paired-end; read1: 26 cycles; i7 index: 8 cycles; read 2: 98 cycles). Raw data were processed using the Cell Ranger pipeline (10× Genomics, v 2.1.0) for cellular barcode assignment and unique molecule identifier (UMI) quantification. From three 10× channels where we loaded the ALL04 and ALL20 cells, we recovered 4402, 5185, and 5542 cells, respectively. Cells with more than 10% UMIs mapped to mitochondrial genes were excluded. After the filtering, the remaining cell numbers are 4291, 5093, and 5385, respectively. Genes with more than 2 UMIs in more than 5% of cells were used for downstream analyses. Expression values for gene *i* in cell *j* were calculated by dividing UMI count values for gene *i* by the sum of the UMI counts in cell *j*, and then multiplying by 10,000 to create transcripts per million (TPM) like values, and finally calculating (TPM + 1) as gene expression values. For comparing single-cell gene expression data, *P* values were calculated using the one-sided Mann–Whitney *U*-test. Uniform Manifold Approximation and Projection (UMAP) clustering of the single-cell RNA sequencing data show the heterogeneity of the transcriptome and the fraction of cells that map to the clonal tracking data (Supplementary Fig. 2).

### Generating and mapping molecular bridges

To specifically amplify molecular bridges containing both a clonal tracking barcode and a cell barcode, we performed PCR using the single-cell cDNA library as the template and a single primer (ACACTCTTTCCCTACACGACGCTCTTCCGATCT). The PCR products were more than 1.5 kb long and were purified from an agarose gel (Zymo Research) before sequencing with the PacBio Sequel sequencer (Pacific Biosciences, v2.1). Raw PacBio sequencing data were analyzed using the circular consensus sequences application of SMRT Analysis software (Pacific Biosciences, SMRT Link Version 5.1.0) with default parameters. Mapping between single-cell gene expression and the clonal activity was established based on the PacBio reads of at least one molecule that contained the exact match to a clonal tracking barcode and a cellular cDNA index. No misread was allowed in this mapping. Cellular cDNA indexes that were mapped to more than one clonal tracking barcodes were excluded from downstream analyses. Around 20% of cells with single-cell transcriptome data were mapped to a tracking barcode.

### False-positive score calculation

A false-positive score (FPS) was calculated by comparing experimental data and scramble data. Five sets of scramble data were generated by randomly mapping tracking barcode data to gene expression data. For each gene, *P* values were calculated by the one-sided Mann–Whitney *U* test using both the experimental data and the scramble data. FPS for each *P* value of the experimental data was then calculated as the number of genes whose *P* values were equal or smaller than this *P* value in the scramble data (median of the five sets) divided by that gene number in the experimental data. Genes with FPS < 0.05 and *P* value < 0.05 were considered significant. Each differential gene expression analysis pooled and compared clones from all experimental mice that received a single patient sample to determine the intratumoral heterogeneity.

### Statistics

The diversity of clones was calculated using the Shannon diversity index, as implemented by Python package skbio.diversity (scikit-bio). K-means clustering method (Fig. 3b) was performed as implemented by python package sklearn.cluster.Kmeans (scikit-learn), the number of clusters was set to 2, and all other parameters were left as default. Tissue biases (Figs. 1i and 2d) were determined based on clonal abundance variations between blood and spleen (99% confidence interval), assuming non-biased clones have equal abundance across tissues. *P* values for clonal response across treatment groups were calculated via an independent *t* test with equal variance assumed. Significance in all figures was indicated as follows: ^***^P < 0.001; ^**^P < 0.01; ^*^P < 0.05; ^n.s.^*P* > 0.05.

### Reporting summary

Further information on research design is available in the Nature Research Reporting Summary linked to this article.

## Supplementary information

Supplementary Information

Reporting Summary

## Data Availability

Raw data of scRNA seq and count matrices are available at the GEO (GSE162506). The remaining data are available in the Article and Supplementary Information. Source data are provided with this paper.

## Source data

Source Data

## Code availability

A custom python script mapping the molecular bridge is available at github.com/dujiang1031/bridge_preprocessing (10.5281/zenodo.5560359). Other python scripts are available upon request.
