# Supplementary material for: Deciphering intratumoral heterogeneity using integrated clonal tracking and single-cell transcriptome analyses
Source: Nat Commun. 2021 Nov 11;12:6522. doi: 10.1038/s41467-021-26771-1 (PMC8586369; doi:10.1038/s41467-021-26771-1)
Supplement: Supplementary file 1 — Supplementary Information [file 41467_2021_26771_MOESM1_ESM.pdf]

Supplementary information for

Deciphering intratumoral heterogeneity using integrated clonal tracking and single-cell transcriptome analyses

Contreras-Trujillo et al.

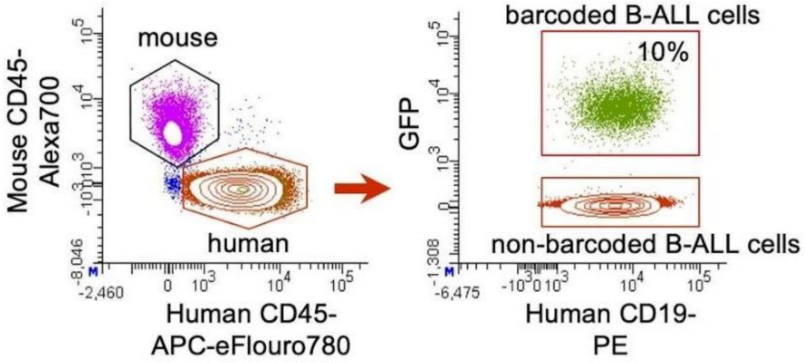

Supplementary Fig. 1 FACS gating for human B-ALL cells in the peripheral blood. Shown is representative peripheral blood analysis of a mouse transplanted with barcoded (GFP+) and unbarcoded (GFP-) B-ALL cells six weeks post transplantation. The gating strategy is applied to all experiments in this study.

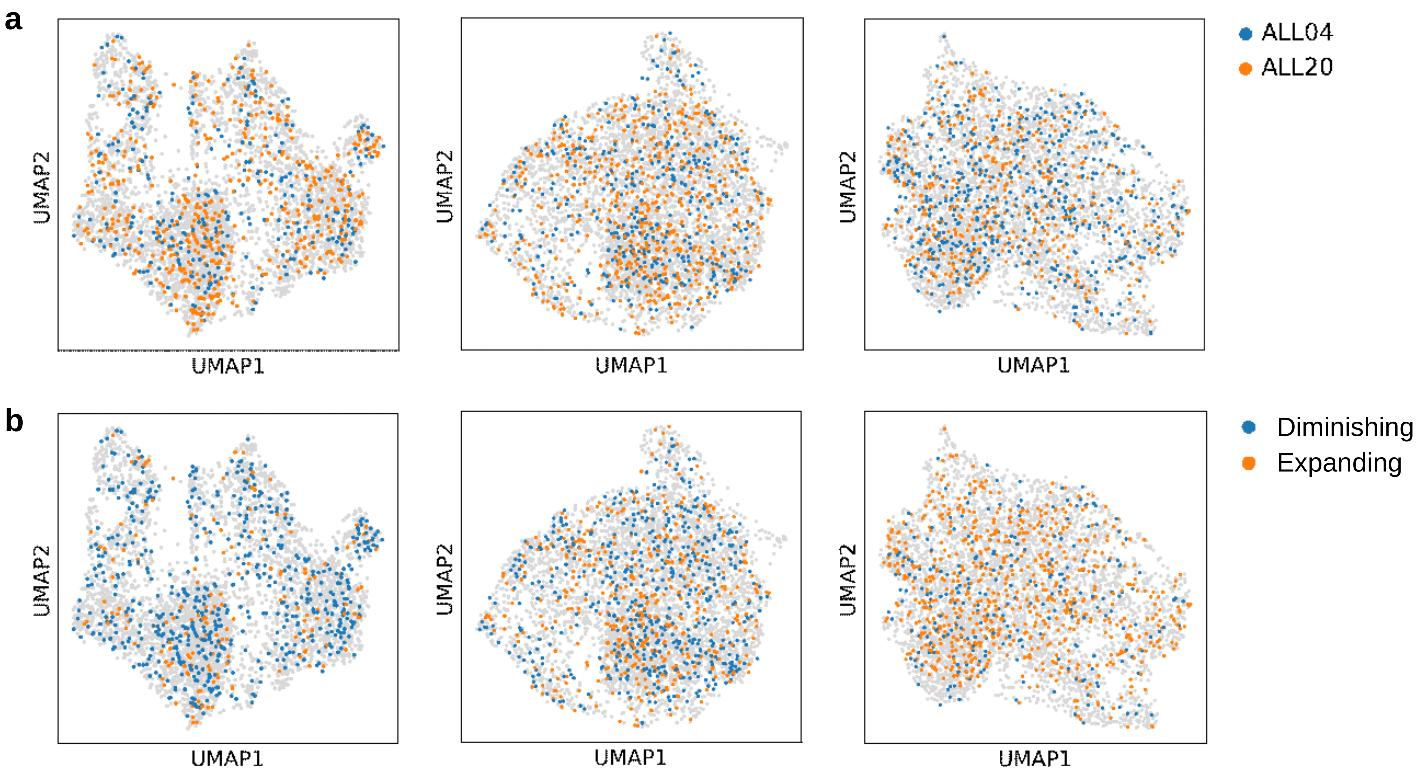

Supplementary Fig. 2 Transcriptome heterogeneity of patient-derived B-ALL cells. UMAP (Uniform Manifold Approximation and Projection) plots showing the scRNA-seq data of human B-ALL cells. Three independent samples from 10X Chromium single cell RNA sequencing analyses are shown. **a** colors highlighted cells derived from patients ALL04 and ALL20. **b** colors highlighted cells mapped to diminishing and expanding clones as classified in Figure 3b.

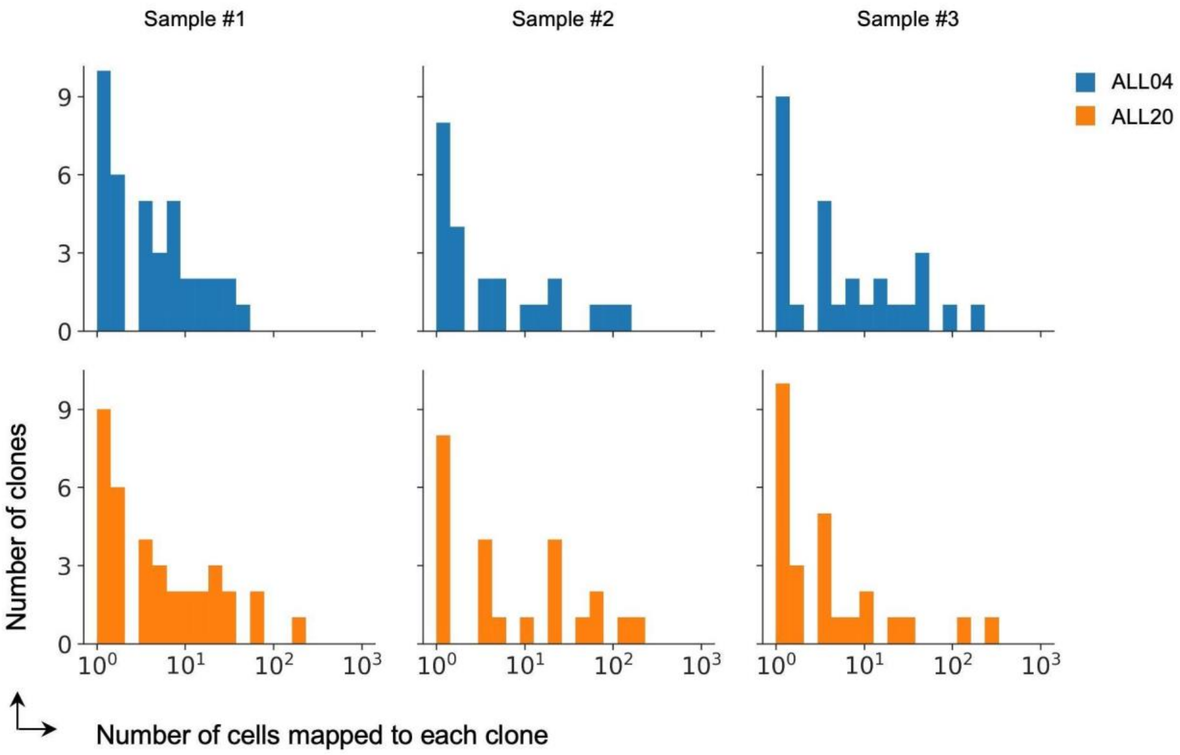

Supplementary Fig. 3 Clone size. Histograms showing the numbers of cells that were mapped to each B-ALL clone derived from patients ALL04 and ALL20.

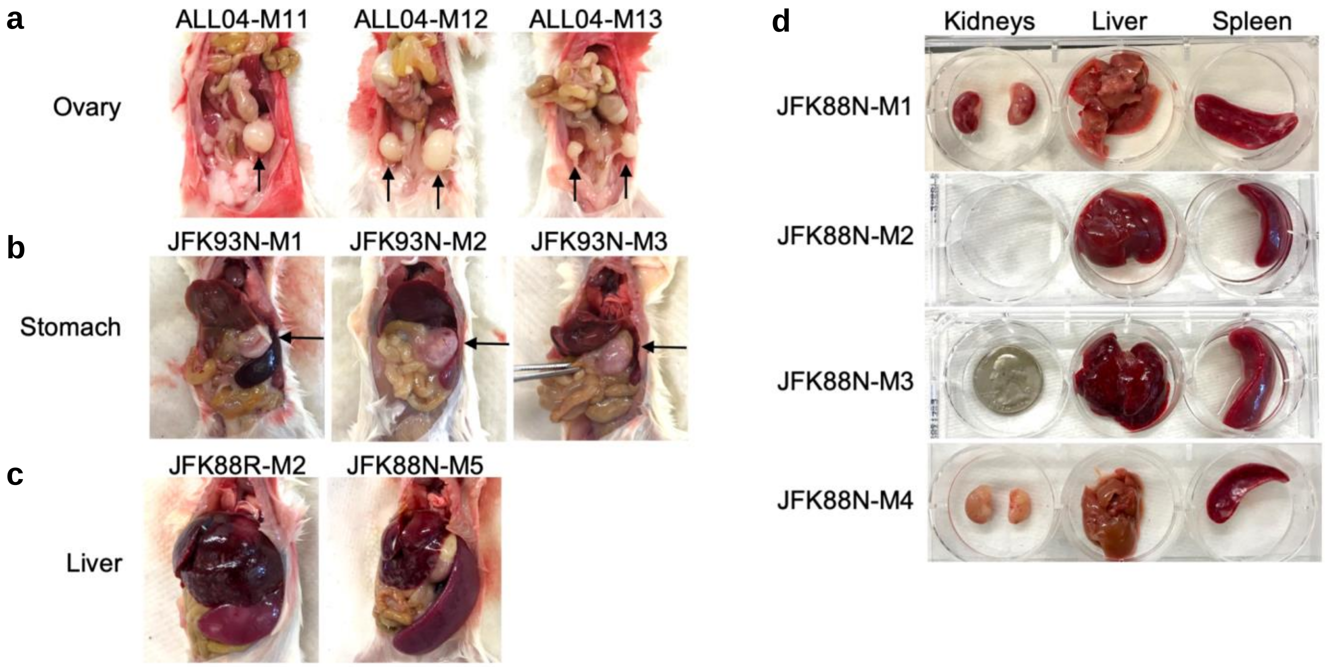

Supplementary Fig. 4 Images of all mice that were xenografted with human B-ALL samples and exhibited enlarged extramedullary organs. a ALL04, **b** JFK93 naïve, **c** JKF88 relapsed, and **d** JFK88 naive. Significant extramedullary expansion was consistently observed in all experimental mice. A quarter is shown as size reference in (**d**).

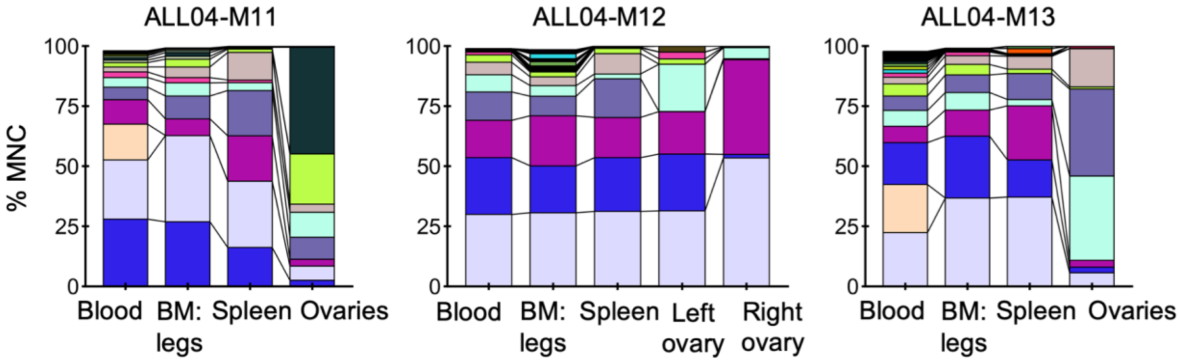

Supplementary Fig. 5 Clonal abundance across different tissues and organs of secondary recipient female mice that were xenografted with the ALL04 sample. Significant extramedullary expansion in the ovaries was observed in all of these mice. Each color represents one distinct genetic barcode corresponding to a leukemia clone. Notably, the examined tissues were saturated with human cells. Spatially confined clonal expansion in the bone marrow was undetectable in these secondary recipient mice, in contrast to the primary recipient mice (Fig. 2c and Supplementary Fig. 4d). Other recipient mice receiving the ALL04 sample, including the primary recipients, were male. Patient ALL04 was also a male (Supplementary Table 1).

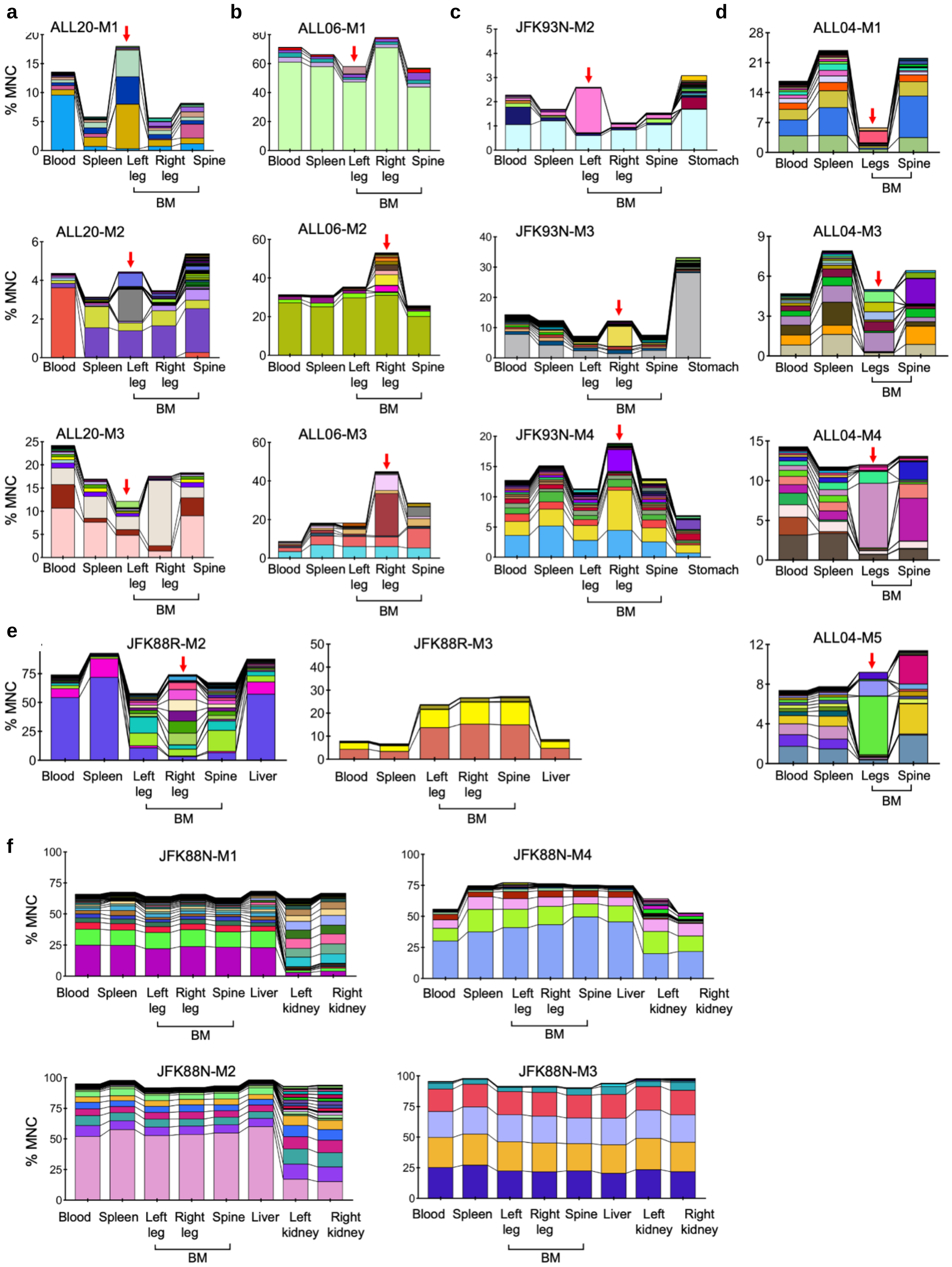

Supplementary Fig. 6 Clonal distribution across different tissues and organs in primary recipient mice. Each color represents one distinct genetic barcode corresponding to a leukemia clone. Red arrows highlight the bone marrow that exhibited different clonal compositions. Shown are all experimental mice, in addition to the one in Fig. 2c, that received the following patient samples: **a** ALL20. **b** ALL06. **c** the naïve stage of patient JFK93. **d** ALL04. **e** the relapsed stage of patient JFK88. **f** the naïve stage of patient JFK88.

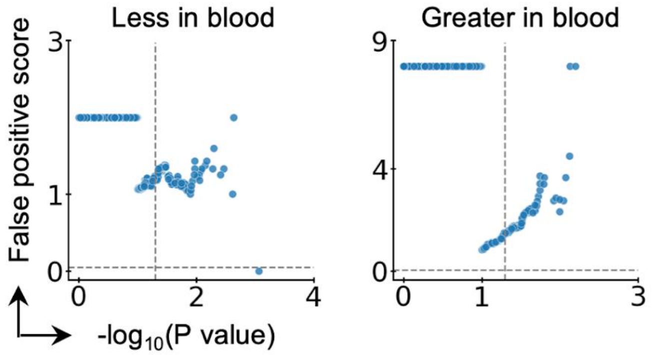

Supplementary Fig. 7 Comparing the gene expression of clones more abundant in the ovary with clones more abundant in the blood (**Fig. 1j**). Shown are False Positive Scores (FPS) and *P* values of differential expression analysis. scRNA-seq data of 4 cells from the clones more abundant in the ovary and 250 cells from the clones more abundant in the blood were used in the analysis. *P* values were calculated by the one-sided Mann Whitney U-test and adjusted using both the experimental data and the scramble data, see the Methods section for detail. Each dot represents a gene. Dashed lines show 0.05 for FPS and *P* values.

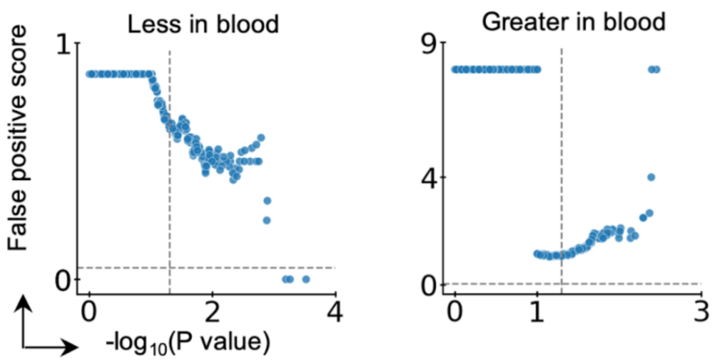

Supplementary Fig. 8 Comparing the gene expression of clones more abundant in the bone marrow with clones more abundant in the blood (Fig. 2e). Shown are False Positive Scores (FPS) and *P* values of differential expression analysis. scRNA-seq data of 9 cells from the clones more abundant in the bone marrow and 98 cells from the clones more abundant in the blood were used in the analysis. *P* values were calculated by the one-sided Mann Whitney U-test and adjusted using both the experimental data and the scramble data, see the Methods section for detail. Each dot represents a gene. Dashed lines show 0.05 for FPS and *P* values.

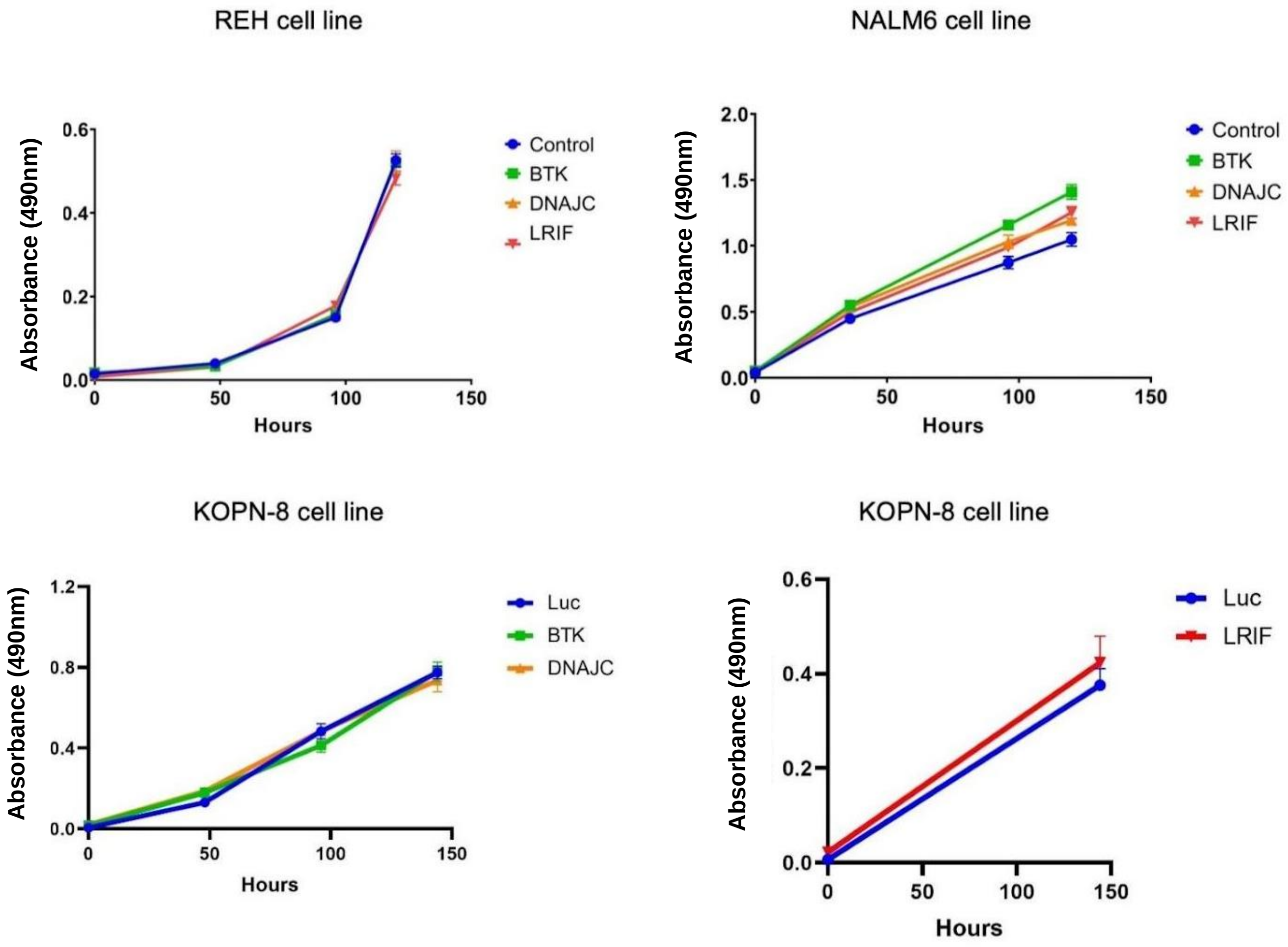

Supplementary Fig. 9 Growth of human B-ALL cells is not altered by the knockout of *BTK, DNAJC*, and *LRIF1* genes. *BTK, DNAJC, LRIF1*, and a negative control luciferase (*LUC*) gene were knocked out using CRISPR/Cas9 technology in human B-ALL cell lines (REH, KOPN-8 and NALM6). The MTS assay was used to quantify viable cells at various time points during the cell culture in complete media. Data from one representative experiment out of three independent experiments are shown. Cell growth was measured in replicates (n=8) at each timepoint. Data shown as mean ± SEM.

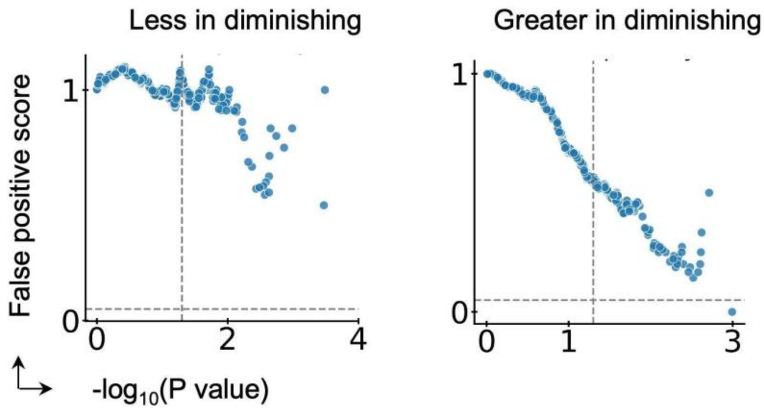

Supplementary Fig. 10 Comparing the gene expression of diminishing clones with expanding clones during serial transplantation (Fig. 3c). Shown are False Positive Scores (FPS) and *P* values of the differential expression analysis. scRNA-seq data of 260 cells from the diminishing clones and 26 cells from the expanding clones were used in the analysis. *P* values were calculated by the one-sided Mann Whitney U-test and adjusted using both the experimental data and the scramble data, see the Methods section for detail. Each dot represents a gene. Dashed lines show 0.05 for FPS and *P* values.

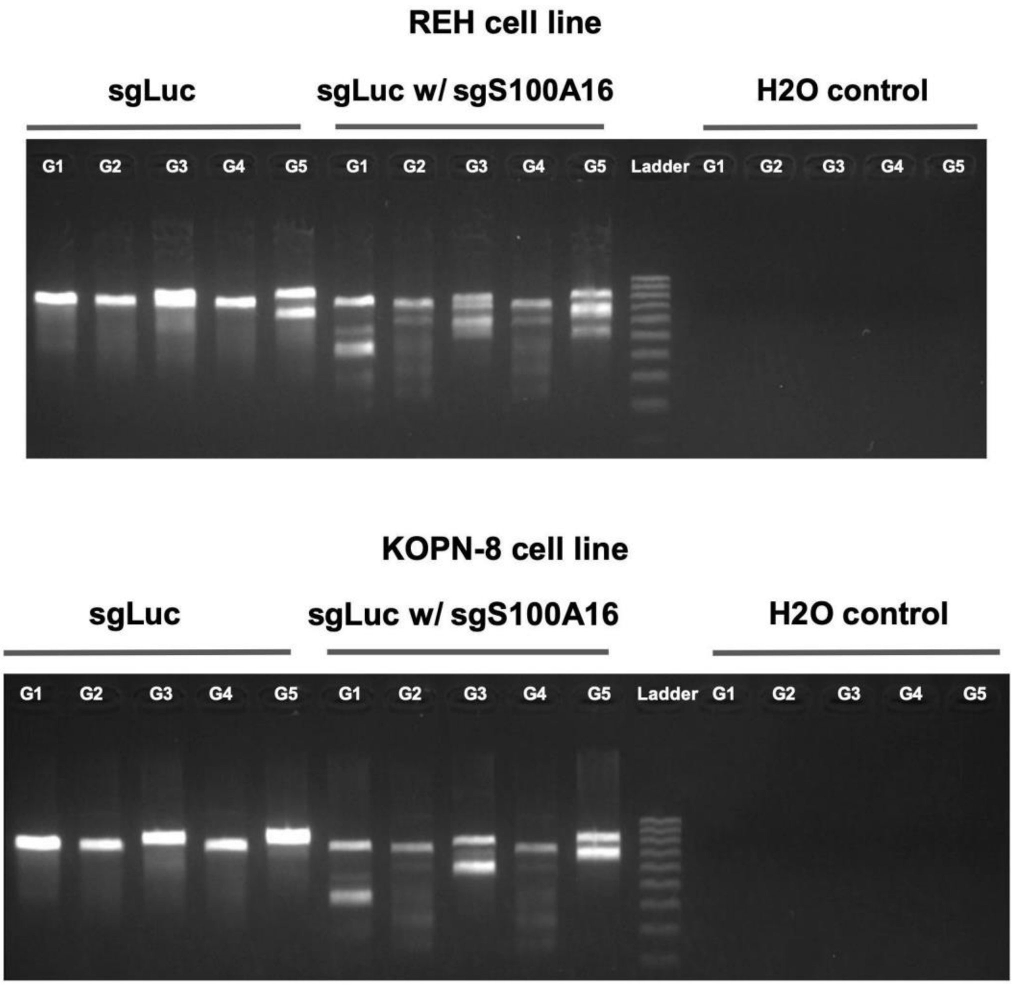

Supplementary Fig. 11 Verifying CRISPR/Cas9 gene knockout by T7E1 assays. Shown are representative gel images of T7E1-treated PCR products amplified from the sgRNA target sites. PCR templates were derived from REH and KOPN-8 cells that were transduced with *Cas9* and pooled sgRNAs targeting negative control luciferase (*LUC*) gene or *S100A16* gene. G1-G5, sgRNAs 1-5. Ladder = 1000bp, 900bp, 800bp, 700bp, 600bp, 500bp, 400bp, 300bp, 200bp, 100bp. Two independent experiments were performed with each experiment using a different cell line. The images depict both results.

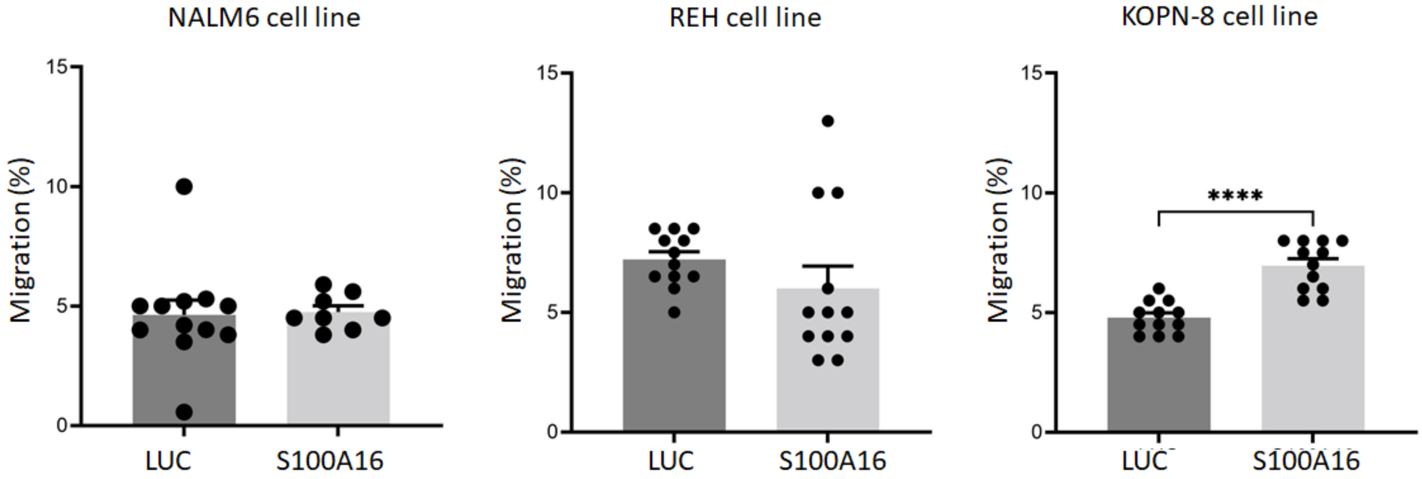

Supplemental Fig. 12. Migration of one out of three B-ALL cell lines is altered by the knockout of *S100A16* gene. *S100A16* and a negative control luciferase (*LUC*) gene were knocked out using CRISPR/Cas9 technology in human B-ALL cell lines (REH, KOPN-8 and NALM6). Migration of the B-ALL cells was analyzed after 12 or 24 hours of incubation (24 hours for REH and KOPN8, 12 hours for NALM6). Three independent experiments (n=12 for REH and KOPN8, n=8 for NALM6). Data shown as mean ± SEM. **** *P* value = 2.85×10^{-6}, by two-sided t-test without adjustment.

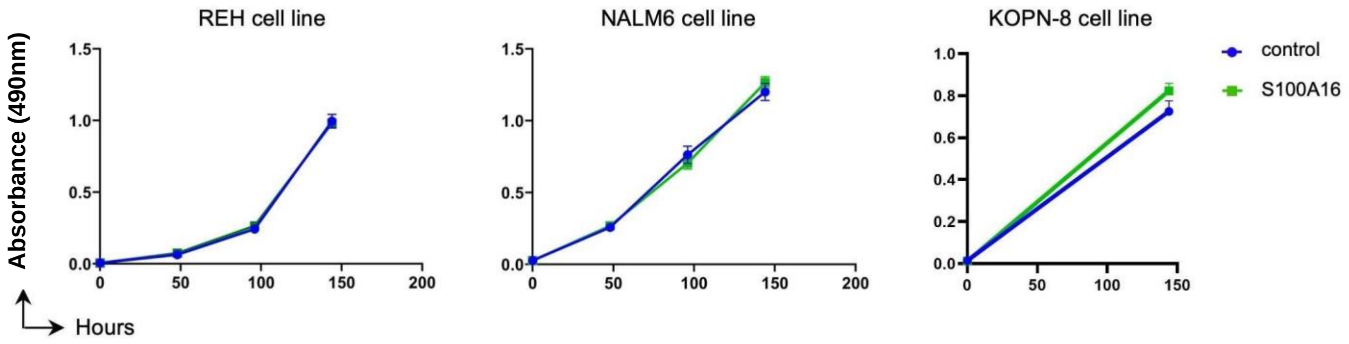

Supplementary Fig. 13 Growth of human B-ALL cells is not altered by the knockout of *S100A16* gene. *S100A16* and a negative control luciferase (*LUC*) gene were knocked out using CRISPR/Cas9 technology in human B-ALL cell lines (REH, KOPN-8 and NALM6). The MTS assay was used to quantify viable cells at various time points during the cell culture in complete media. Data from one representative experiment out of three independent experiments are shown. Cell growth was measured in replicates (n=8) at each timepoint. Data shown as mean ± SEM.

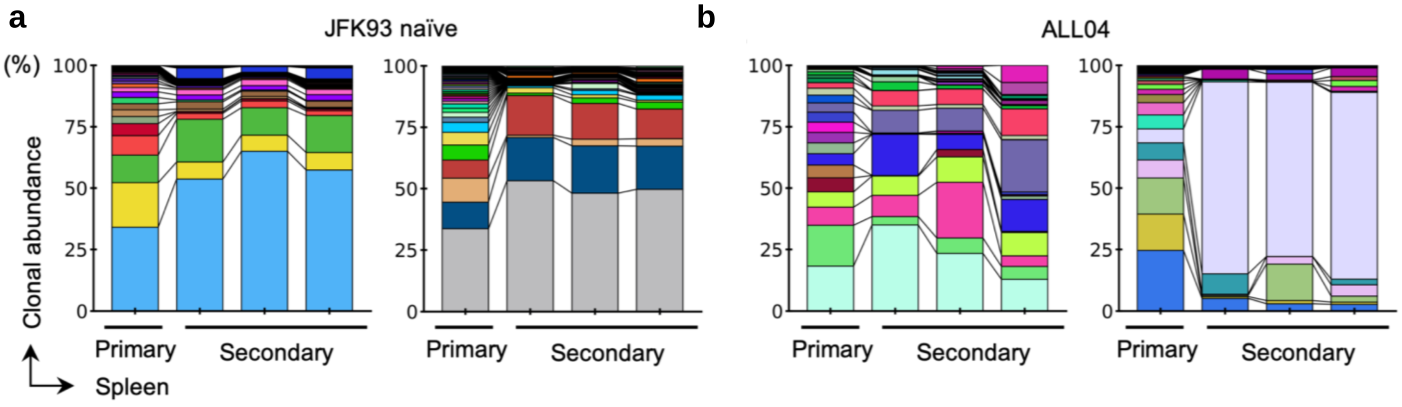

Supplementary Fig. 14 Clonal abundance changes between primary and secondary recipients. Data were collected from the spleen and normalized among barcoded cells. Each column shows data from one mouse. Four independent experiments were performed using samples from two patients, **a** JFK93 and **b** ALL04. Each color represents one distinct genetic barcode corresponding to a leukemia clone.

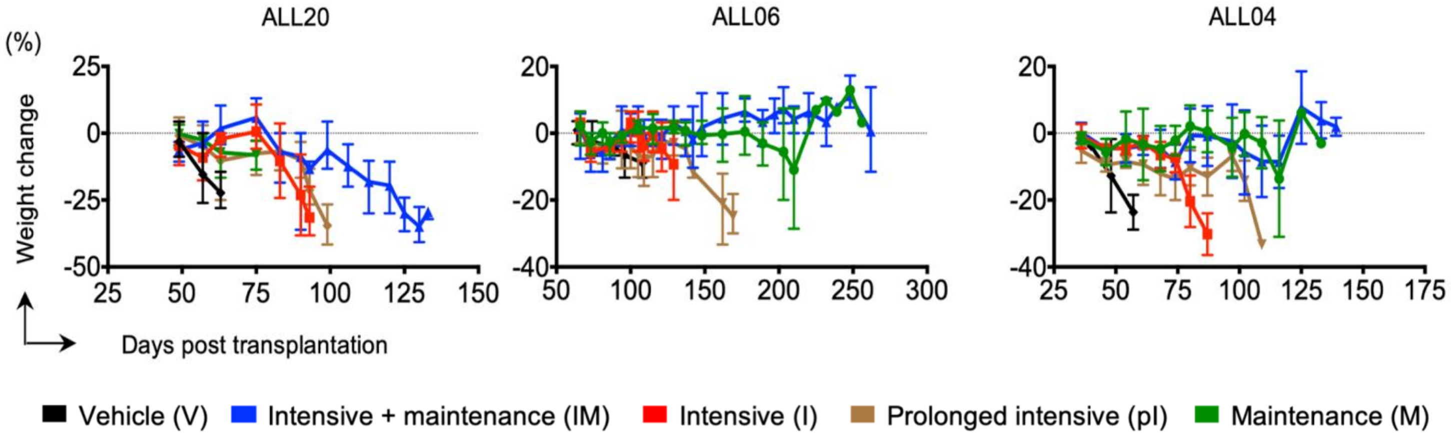

Supplementary Fig. 15 Mouse body weight before and during chemotherapy treatments. Shown are the mean of all experimental mice. Error bar represents range. Biological replicate number (mouse number) is as follows. ALL20: n=13 V, n=11 IM, n=8 I, n=10 pI, and n=5 M. ALL06: n=4 V, n=5 IM, n=3 I, n=4 pI, and n=4 M. ALL04: n=4 for each experimental group.

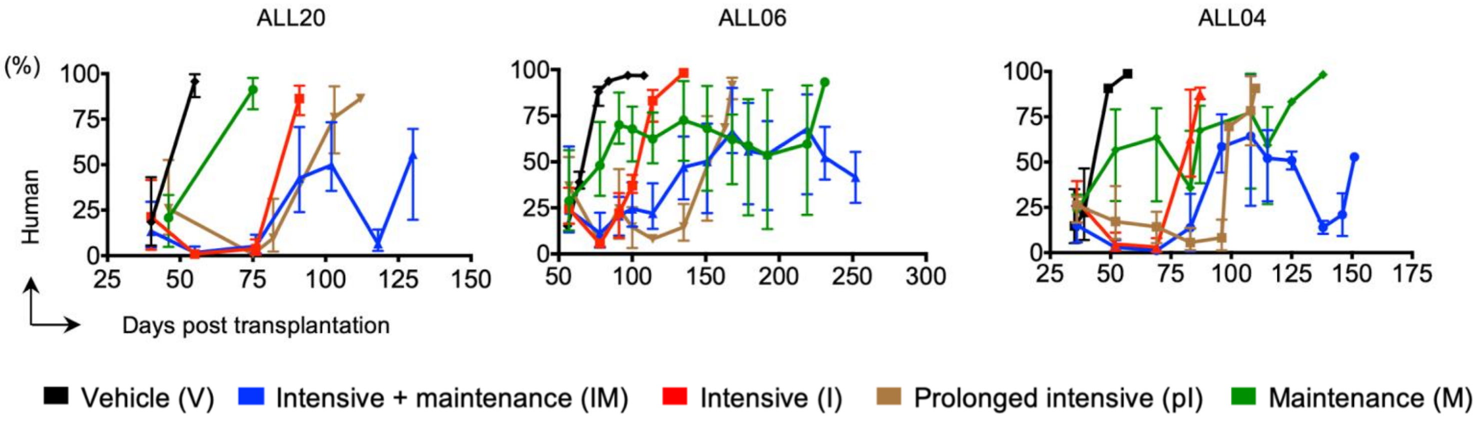

Supplementary Fig. 16 Human chimerism in the peripheral blood before and during chemotherapy treatments. Shown are the mean of all experimental mice. Error bar represents range. Biological replicate number (mouse number) is as follows. ALL20: n=13 V, n=11 IM, n=8 I, n=10 pI, and n=5 M. ALL06: n=4 V, n=5 IM, n=3 I, n=4 pI, and n=4 M. ALL04: n=4 for each experimental group.

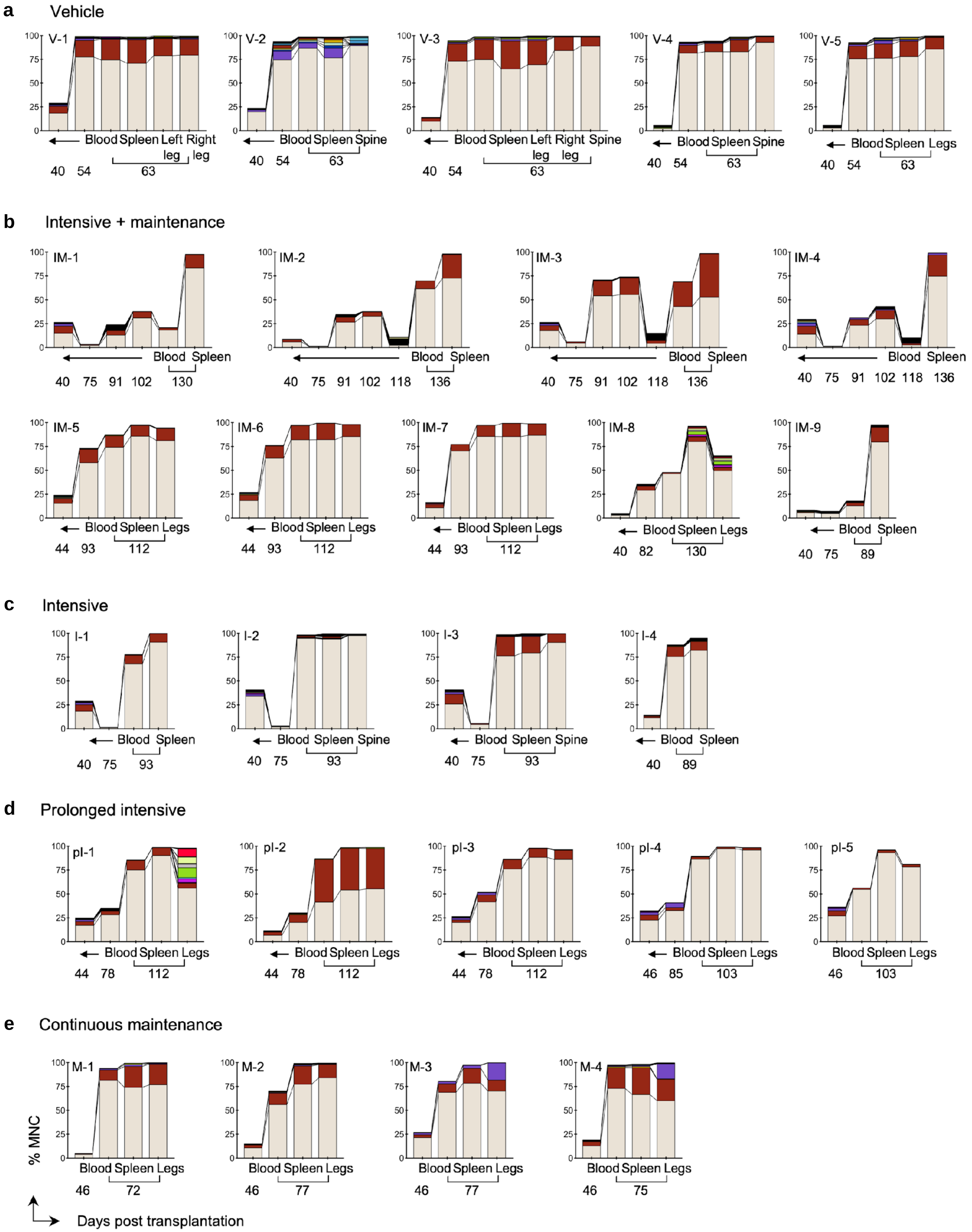

Supplementary Fig. 17 Clonal dynamics during chemotherapy treatments in mice that received B-ALL cells from patient ALL20. Shown are clonal abundances of all experimental mice during the chemotherapy treatments as follows: **a** vehicle; **b** intensive and maintenance; **c** intensive; **d** prolonged intensive; and **e** maintenance. Each color represents one distinct genetic barcode corresponding to a leukemia clone.

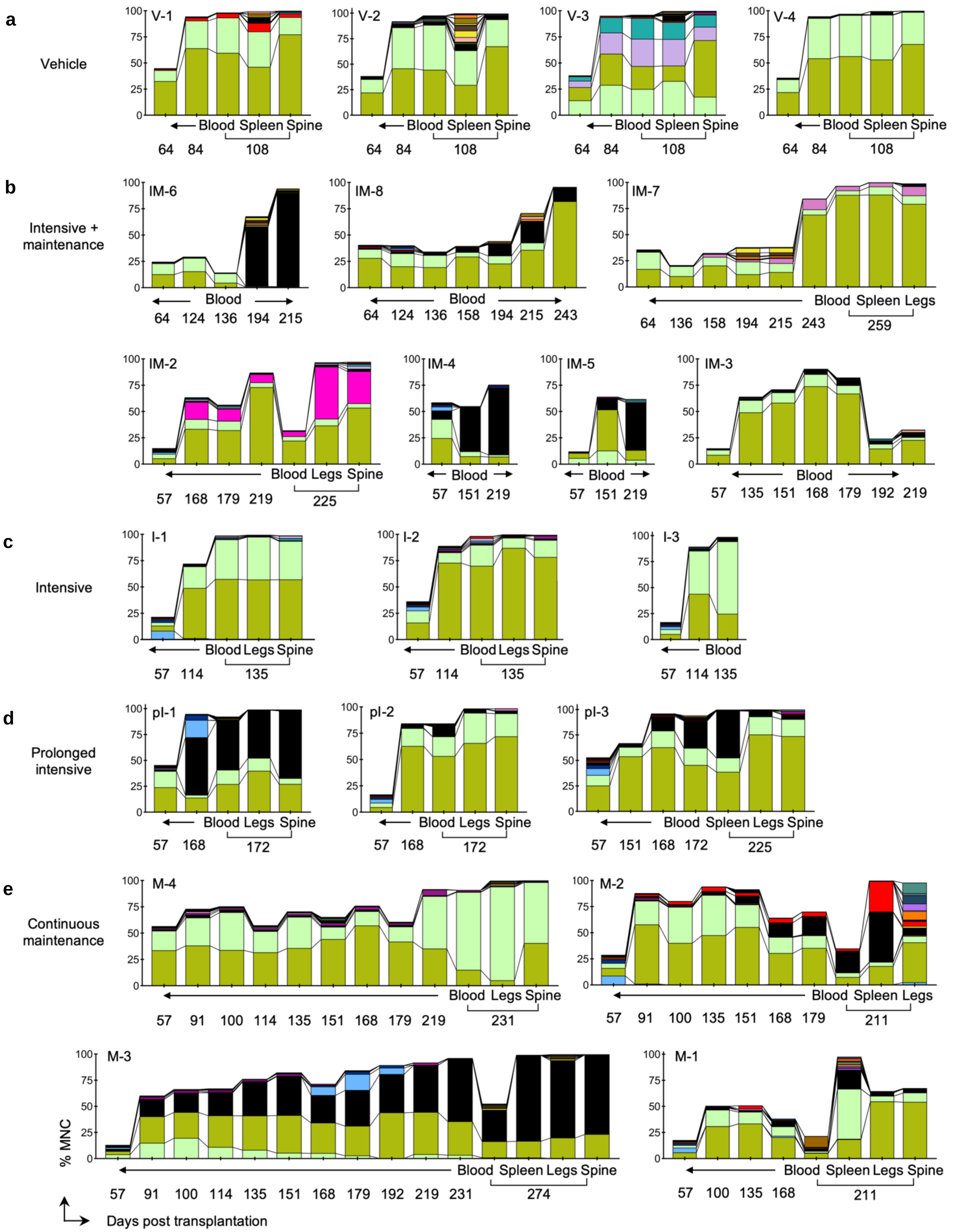

Supplementary Fig. 18 Clonal dynamics during chemotherapy treatments in mice that received B-ALL cells from patient ALL06. Shown are clonal abundances of all experimental mice during the chemotherapy treatments as follows: **a** vehicle; **b** intensive and maintenance; **c** intensive; **d** prolonged intensive; and **e** maintenance. Each color represents one distinct genetic barcode corresponding to a leukemia clone.

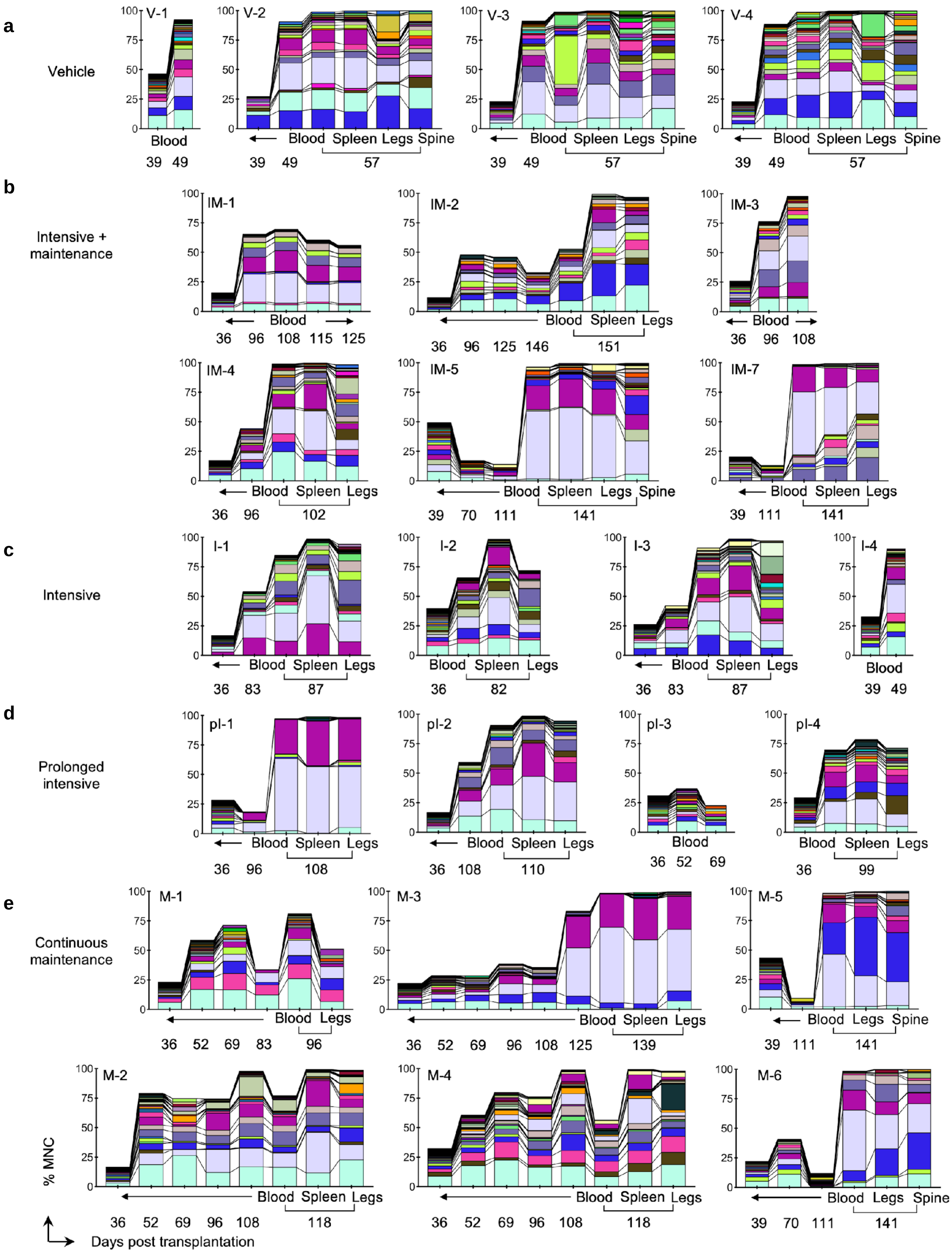

Supplementary Fig. 19 Clonal dynamics during chemotherapy treatments in mice that received B-ALL cells from patient ALL04. Shown are clonal abundances of all experimental mice during the chemotherapy treatments as follows: **a** vehicle; **b** intensive and maintenance; **c** intensive; **d** prolonged intensive; and **e** maintenance. Each color represents one distinct genetic barcode corresponding to a leukemia clone.

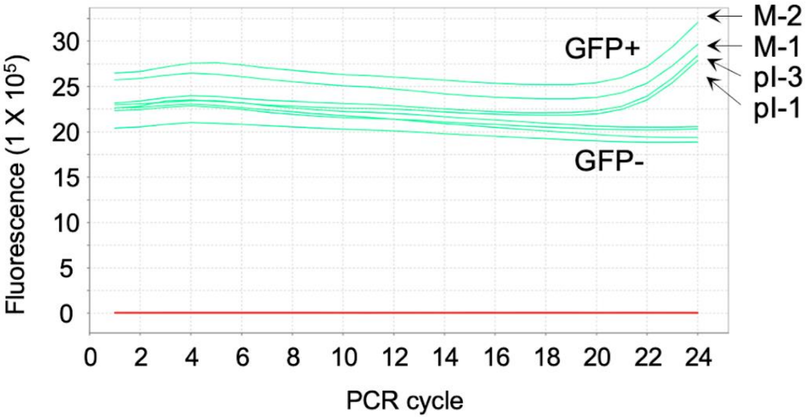

Supplementary Fig. 20 qPCR analysis of GFP negative clones emerging during chemotherapy treatments from mice that received B-ALL cells of patient ALL06. Shown are amplification curves from sorted GFP negative (GFP-) cells. Four GFP positive (GFP+) samples from different treatment groups are included as positive controls.

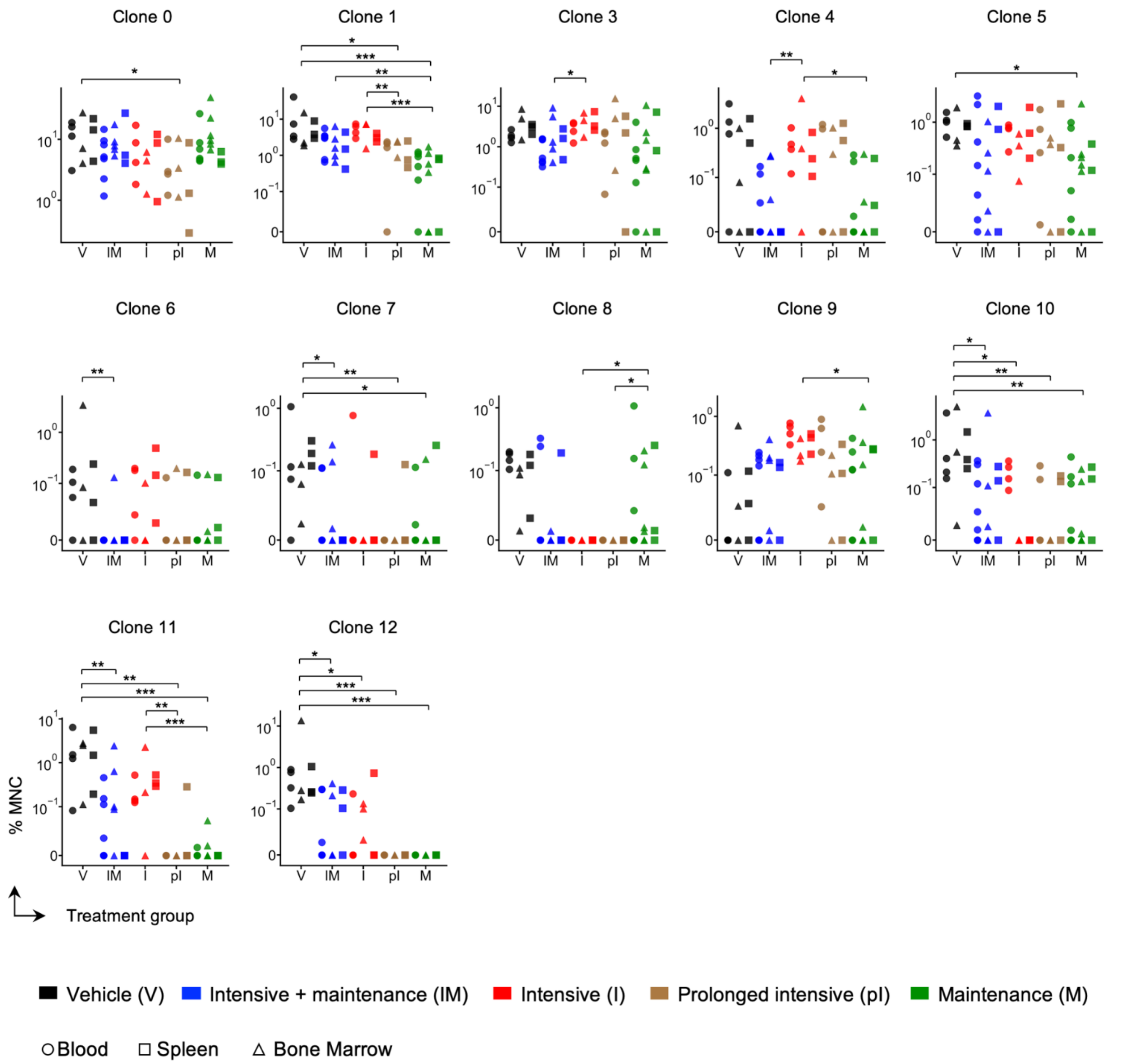

Supplementary Fig. 21 ALL04 clones that responded significantly differently to different chemo treatments. Each plot represents data from one clone, and each marker represents data from one tissue and one mouse. Data from 25 mice were used in each graph. MNC - mononuclear cells; *** $P < 0.001$; ** $P < 0.01$; * $P < 0.05$. P values were calculated by two-sided Kruskal Wallace test with Bonferroni correction.

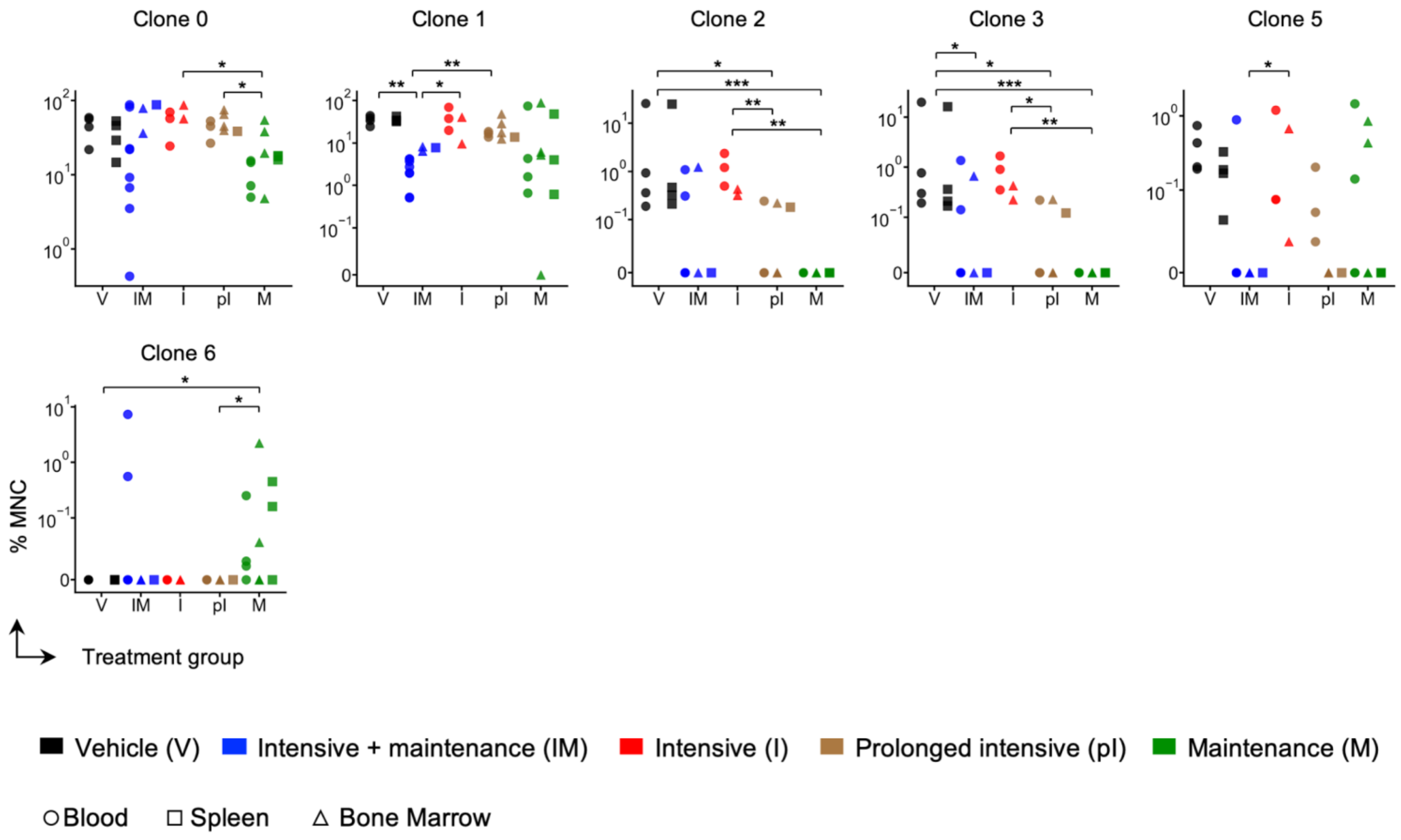

Supplementary Fig. 22 ALL06 clones that responded significantly differently to different chemo treatments. Each plot represents data from one clone, and each marker represents data from one tissue and one mouse. Data from 23 mice were used in each graph. MNC - mononuclear cells; *** $P < 0.001$; ** $P < 0.01$; * $P < 0.05$. P values were calculated by two-sided Kruskal Wallace test with Bonferroni correction.

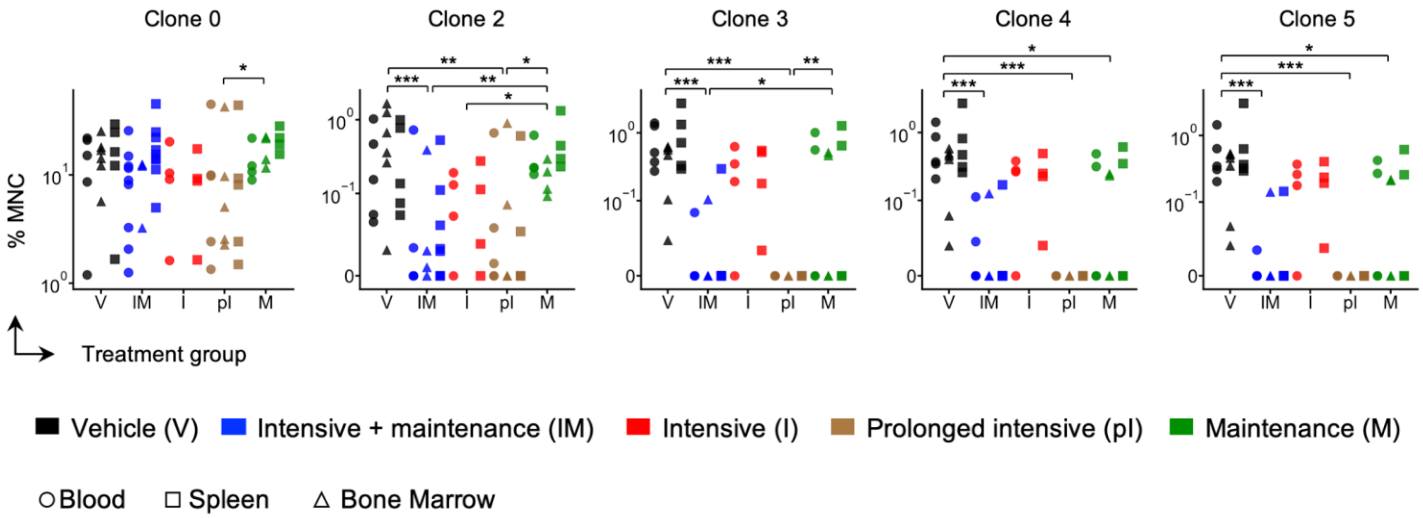

Supplementary Fig. 23 ALL20 clones that responded significantly differently to different chemo treatments. Each plot represents data from one clone, and each marker represents data from one tissue and one mouse. Data from 50 mice and 49 mice were used for Clone 0 and Clone 2 respectively, and data from 27 mice were used in the remaining graphs, as some clones did not engraft in every mouse. MNC - mononuclear cells; *** $P < 0.001$; ** $P < 0.01$; * $P < 0.05$. P values were calculated by two-sided Kruskal Wallace test with Bonferroni correction.

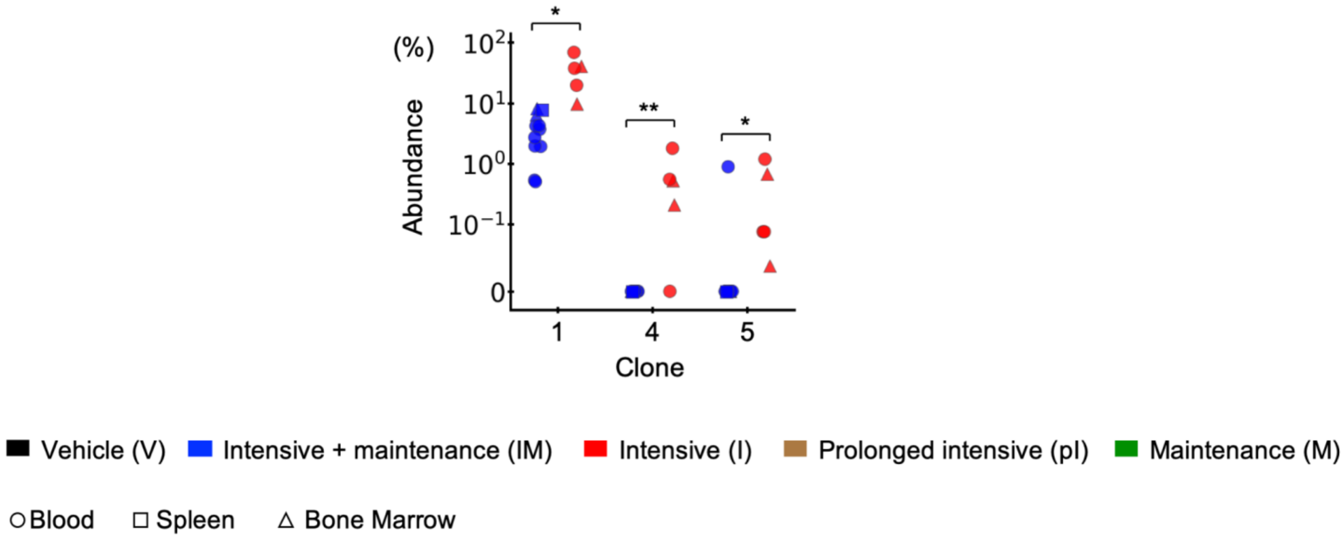

Supplementary Fig. 24 ALL06 clones that responded significantly better to combination therapy than to intensive therapy. Each marker represents data from one tissue and one mouse. Data from 23 mice were used in each graph . ** $P < 0.01$; * $P < 0.05$. P values were calculated by two-sided Kruskal Wallace test with Bonferroni correction.

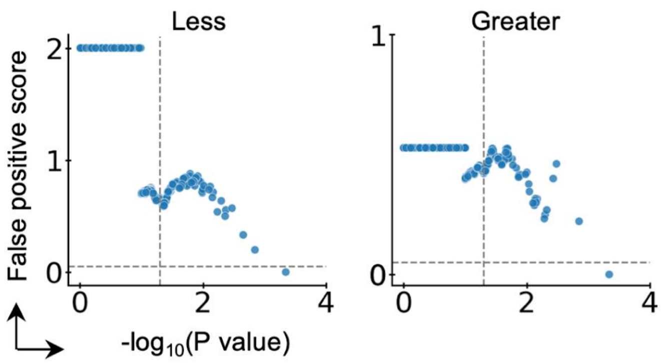

Supplementary Fig. 25 Comparing the gene expression of ALL04 clones that responded significantly better to combination therapy than to intensive therapy with all other ALL04 clones (Fig. 5c). Shown are False Positive Scores (FPS) and *P* values of differential expression analysis. scRNA-seq data of 41 cells from the selected clones and 958 cells from the rest of the clones were used in the analysis. *P* values were calculated by the one-sided Mann Whitney U-test and adjusted using both the experimental data and the scramble data, see the Methods section for detail. Each dot represents a gene. Dashed lines show 0.05 for FPS and *P* values.

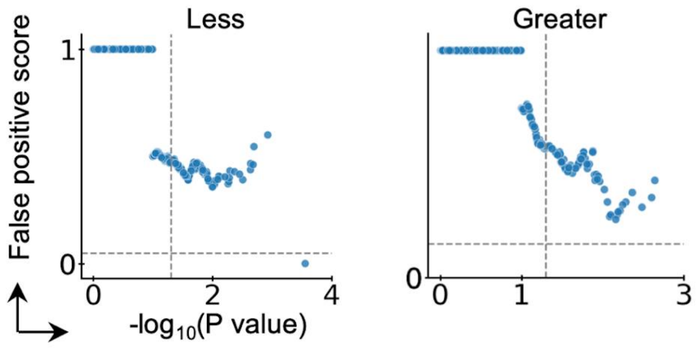

Supplementary Fig. 26 Comparing the gene expression of ALL04 clones that responded better to maintenance therapy than to intensive therapy with all other ALL04 clones (Fig. 5d). Shown are False Positive Scores (FPS) and *P* values of differential expression analysis. scRNA-seq data of 97 cells from the selected clones and 902 cells from the rest of the clones were used in the analysis. *P* values were calculated by the one-sided Mann Whitney U-test and adjusted using both the experimental data and the scramble data, see the Methods section for detail. Each dot represents a gene. Dashed lines show 0.05 for FPS and *P* values.

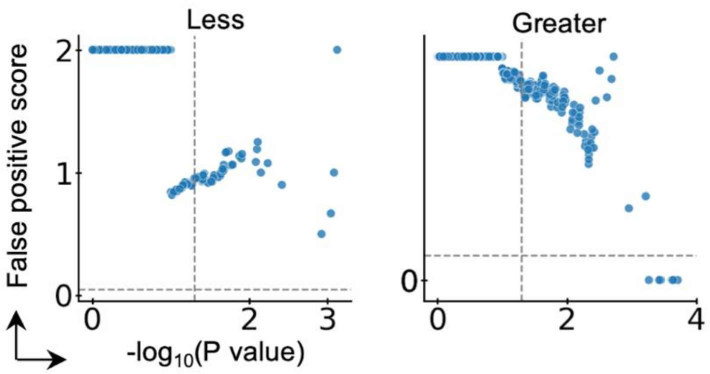

Supplementary Fig. 27 Comparing the gene expression of ALL20 clones that responded better to intensive therapy than to maintenance therapy with all other ALL20 clones (Fig. 5e). Shown are False Positive Scores (FPS) and *P* values of differential expression analysis. scRNA-seq data of 63 cells from the selected clones and 1038 cells from the rest of the clones were used in the analysis. Each dot represents a gene. *P* values were calculated by the one-sided Mann Whitney U-test and adjusted using both the experimental data and the scramble data, see the Methods section for detail. Dashed lines show 0.05 for FPS and *P* values.

a

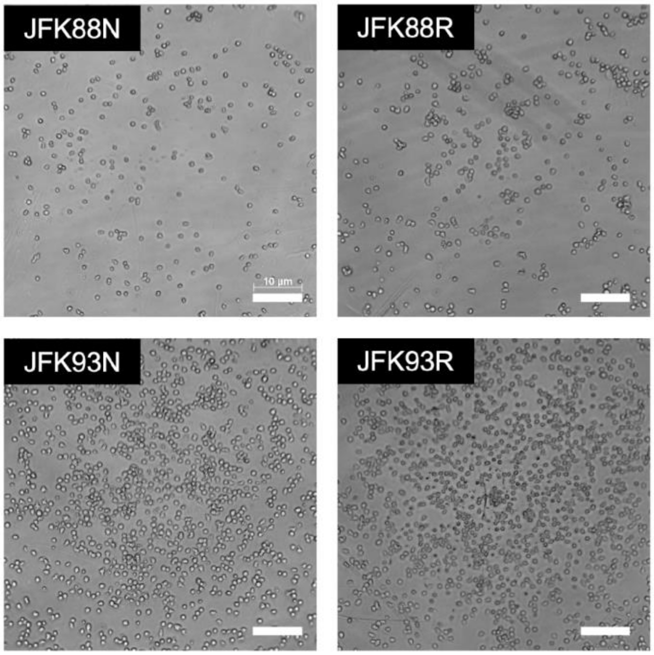

b

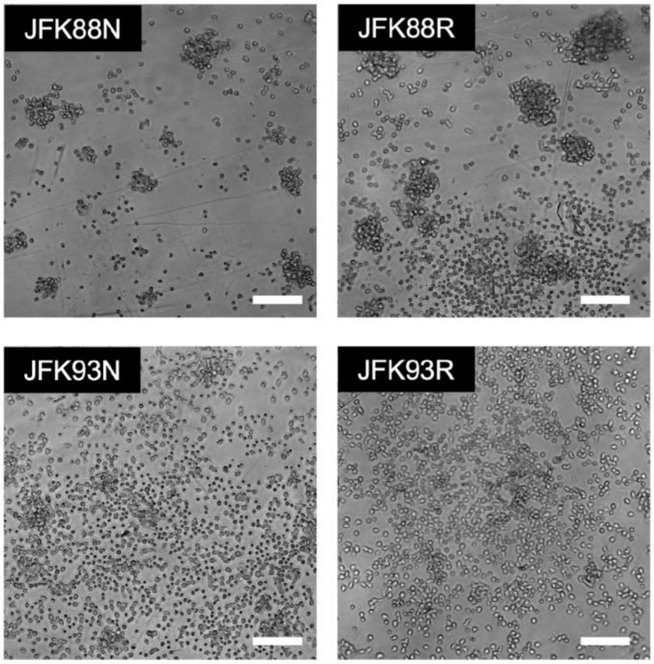

Supplementary Fig. 28 *In vitro* culture of B-ALL cells. Equal numbers of leukemia cells acquired at different disease stages, naïve (N) and relapsed (R), from two patients (JFK88 and JFK93) were cultured *in vitro* during the lentiviral transduction. **a** 24-hours after plating. **b** 48-hours after plating right before transplantation. White bar represents a 10 µm scale bar. Representative images are shown from an experiment with five replicates per sample.

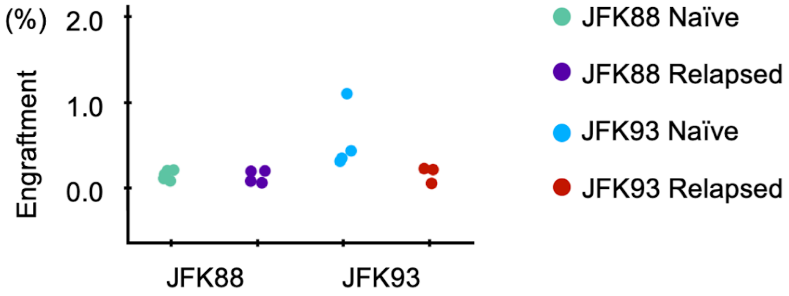

Supplementary Fig. 29 Engraftment of human B-ALL cells. The engraftment rate was calculated based on the number of donor cells and the number of clones detected in the peripheral blood, bone marrow, spleen and enlarged extramedullary organs, adjusted for GFP%. Each dot represents data from one mouse.

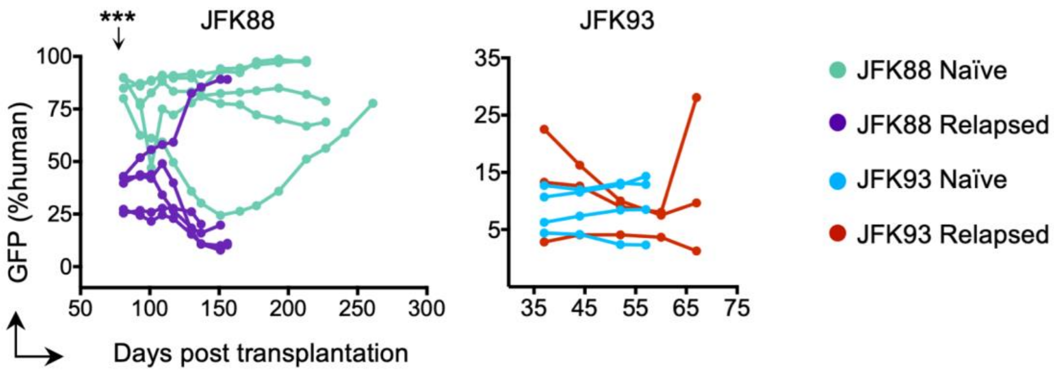

Supplementary Fig. 30 Transduction of human B-ALL cells. Each line represents data from one recipient mouse. An unpaired two-tailed t-test was performed to measure the statistical significance for JFK88 at day 81 post transplantation. ****P* value = 0.000015.

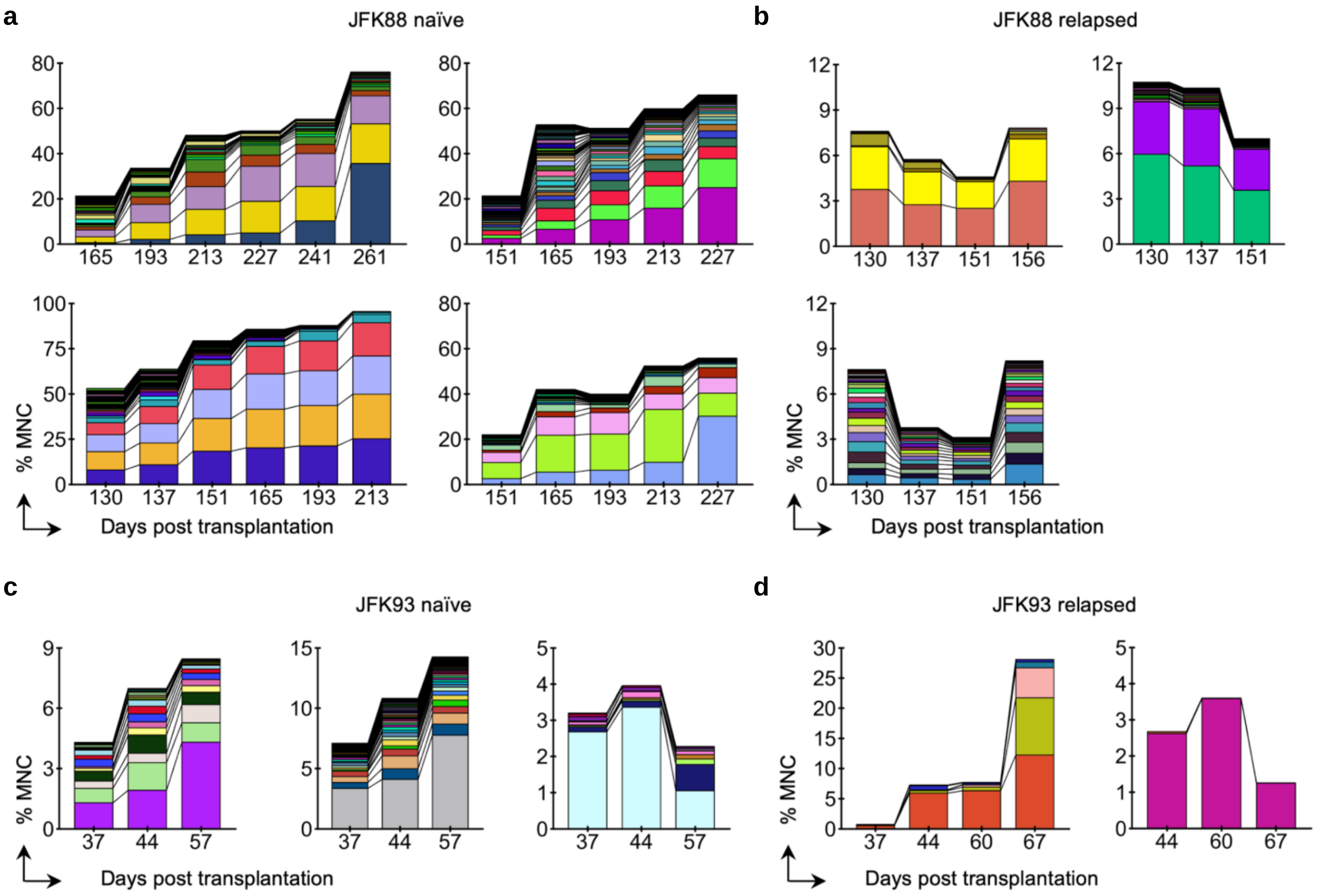

Supplementary Fig. 31 Clonal dynamics of human B-ALL cells in PDX model. Shown are all experimental mice in addition to those in Fig. 6f that received naïve (**a, c)** and relapsed samples (**b, d)** of patient JFK88 and JFK93. Each color represents one distinct genetic barcode corresponding to a leukemia clone. Data were collected from the peripheral blood and normalized among all mononuclear cells (MNC).

Supplementary Table 1. Patient sample information.

Patient ID	BM_mnc_bl	PB_mnc_bl	Diagnosis	Flow cytometry immunophenotyping results at diagnosis	Karyotype Description	Molecular Description
JFK086	**85%**	**59%**	Early Pre-B ALL (mu chains not tested)	Peripheral Blood:<br>CD9+++, CD10+, CD19+<br>CD20-,CD22weak, CD24++<br>CD34-, CD38+++<br><br>GPR56+	45,XX,del(9)(p21),-20[11]/<br>46,XX,del(9),del(20)(q11.1)[8]	[RNA# 26038BM]<br>[BCR/ABLNEG]<br>[MLL/AF4NEG] [TEL/AML1NEG]<br>[E2A/PBX1NEG]<br>[B-other phenotype as per C. Mullighan]
JFK088	**71%**	**47%**	Early Pre-B ALL (mu chains not tested)	Peripheral Blood:<br>CD9variably pos, CD10++<br>CD20 about 20%, CD22weak, CD24++, CD25-<br>CD34-, CD38+, CD90-<br>CD33+CD13 neg<br>CD15+CD65neg,<br><br>GPR56-, CRLF2-	46,XX [20]	[RNA# 25152BM]<br>[BCR/ABLNEG]<br>[MLL/AF4NEG] [TEL/AML1NEG]<br>[E2A/PBX1NEG]<br>[B-other phenotype as per C. Mullighan]
JFK093	**98%**	**55% rest T-lys**	Early Pre-B ALL (mu chains not tested)	Peripheral Blood:<br>CD9++,CD10++<br>CD20+,CD24++,<br>CD34[25%], CD38+,<br>GPR56++, CRLF2++	46,XX [20]	[RNA# 26498BM]<br>[BCR/ABLNEG]<br>[Ph-like_CRLF2 phenotype with IGH-CRLF2 kinase alteration as per C. Mullighan]
ALL04	**n/a**	**n/a**	Pre-B ALL	Bone Marrow:<br>**Lymphoid population**: 1% of total events<br>**Monocyte population**: 0.2% of total events<br>**Myeloid population**: 1% of total events<br>**Blast population**: 97% of total events and express: bright CD10, CD19, HLA-DR; weak CD20, FMC7, CD38 and are negative for KAPPA, LAMBDA.	n/a	FISH Results:<br>Negative for t(9:22) BCR/ABL
ALL06	**n/a**	**n/a**	Pre-B ALL	Peripheral Blood:<br>**Lymphoid population:** 28% of total events. These include B-Cells (24%) with a kappa:lamda ration of 1:1, T-cells (76%) with a CD4:CD8 ratio of 0.8, and NK cells (0%).<br>B-cells express CD19, CD20, variable CD23, variable FMC-7. T cells express CD2, CD3, CD5, variable CD7 and either CD4 or CD8 without loss of pan-T cell marker<br>**Monocyte population**: <1% of total events and exress CD4, CD14, CD16, CD64, CD13, CD33<br>**Myeloid population:** 3% of total events. They express CD10, CD16, CD13, CD33 without expression of CD34 and CD117.<br>**Blast population**: 52% of total events and express: CD10 (bright), CD19 (dim), CD20 (dim to moderate), FMC7 (dim to moderate), CD38 CD71 (dim) HLA-DR (heterogeneous. A subset of blast (31%) express CD34. The blasts are negative for myeloid associated antigens.	46 XY [10]<br>normal karyotype, no evidence of clonal abnormality	FISH Results:<br>Negatice for MLL,<br>Negative for t(9:22) BCR/ABL
ALL20	**n/a**	**n/a**	Pre-B ALL	Peripheral Blood:<br>94% blast with modertate CD19, bright CD10, dim CD20, variable CD34, moderate CD38, bright HLA-DR, and lack of light chains.	46, XY [15]	FISH Results:<br>Negatice for BCR/ABL

Supplementary Table 2. Monoclonal antibodies used in this study.

Antigen	Conjugate	Vendor	Catalog #	Clone	Lot #	Dilution
mouse CD45	Alexa 700	Biolegend	110724	A20	B254605	1:100
human CD45	APC-eFluor 780	eBioscience	47-0459-42	H130	4331639	1:100
human CD19	PE	eBioscience	12-0198-42	SJ25C1	1987712	1:100
